# Genome-wide association and epidemiological analyses reveal common genetic origins between uterine leiomyomata and endometriosis

**DOI:** 10.1038/s41467-019-12536-4

**Published:** 2019-10-24

**Authors:** C. S. Gallagher, N. Mäkinen, H. R. Harris, N. Rahmioglu, O. Uimari, J. P. Cook, N. Shigesi, T. Ferreira, D. R. Velez-Edwards, T. L. Edwards, S. Mortlock, Z. Ruhioglu, F. Day, C. M. Becker, V. Karhunen, H. Martikainen, M.-R. Järvelin, R. M. Cantor, P. M. Ridker, K. L. Terry, J. E. Buring, S. D. Gordon, S. E. Medland, G. W. Montgomery, D. R. Nyholt, D. A. Hinds, J. Y. Tung, the 23andMe Research Team, Michelle Agee, Babak Alipanahi, Adam Auton, Robert K. Bell, Katarzyna Bryc, Sarah L. Elson, Pierre Fontanillas, Nicholas A. Furlotte, Karen E. Huber, Aaron Kleinman, Nadia K. Litterman, Matthew H. McIntyre, Joanna L. Mountain, Elizabeth S. Noblin, Carrie A. M. Northover, Steven J. Pitts, J. Fah Sathirapongsasuti, Olga V. Sazonova, Janie F. Shelton, Suyash Shringarpure, Chao Tian, Vladimir Vacic, Catherine H. Wilson, J. R. B. Perry, P. A. Lind, J. N. Painter, N. G. Martin, A. P. Morris, D. I. Chasman, S. A. Missmer, K. T. Zondervan, C. C. Morton

**Affiliations:** 1 Department of Genetics, Harvard Medical School, Boston, MA 02115 USA; 2 Department of Obstetrics and Gynecology, Brigham and Women’s Hospital, Harvard Medical School, Boston, MA 02115 USA; 3 Program in Epidemiology, Division of Public Health Sciences, Fred Hutchinson Cancer Research Center, Seattle, WA 98109 USA; 4 Wellcome Centre for Human Genetics, University of Oxford, Oxford, OX3 7BN UK; 5 Endometriosis CaRe Centre, Nuffield Department of Women’s and Reproductive Health, University of Oxford, John Radcliffe Hospital, Oxford, OX3 9DU UK; 6 Department of Obstetrics and Gynecology, Oulu University Hospital and PEDEGO Research Unit & Medical Research Center Oulu, University of Oulu and Oulu University Hospital, 90220 Oulu, Finland; 7 Department of Biostatistics, University of Liverpool, Liverpool, L69 3GL UK; 8 Big Data Institute, Li Ka Shing Center for Health Information and Discovery, Oxford University, Oxford, OX3 7LF UK; 9 Vanderbilt Genetics Institute, Vanderbilt Epidemiology Center, Institute for Medicine and Public Health, Department of Obstetrics and Gynecology, Vanderbilt University Medical Center, Nashville, TN 37203 USA; 10 Division of Epidemiology, Department of Medicine, Institute for Medicine and Public Health, Vanderbilt Genetics Institute, Vanderbilt University Medical Center, Nashville, TN 37203 USA; 11 Institute for Molecular Bioscience, University of Queensland, Brisbane, QLD 4072 Australia; 12 MRC Epidemiology Unit, University of Cambridge School of Clinical Medicine, Institute of Metabolic Science, Cambridge Biomedical Campus, Cambridge, CB2 0QQ UK; 13 Center for Life Course Health Research, Faculty of Medicine, University of Oulu, 90220 Oulu, Finland; 14 Unit of Primary Health Care, Oulu University Hospital, 90220 Oulu, Finland; 15 Department of Epidemiology and Biostatistics, MRC-PHE Centre for Environment and Health, School of Public Health, Imperial College London, London, W2 1PG UK; 16 Biocenter Oulu, University of Oulu, 90220 Oulu, Finland; 17 Department of Life Sciences, College of Health and Life Sciences, Brunel University London, Uxbridge, Middlesex UB8 3PH UK; 18 Department of Human Genetics, David Geffen School of Medicine, University of California at Los Angeles, Los Angeles, CA 90095 USA; 19 Division of Preventative Medicine, Brigham and Women’s Hospital, Harvard Medical School, Boston, MA USA; 20 Obstetrics and Gynecology Epidemiology Center, Brigham and Women’s Hospital and Harvard Medical School, Boston, MA 02115 USA; 21 Department of Epidemiology, Harvard T.H. Chan School of Public Health, Boston, MA 02115 USA; 22 Genetic Epidemiology, QIMR Berghofer Medical Research Institute, Brisbane, QLD 4006 Australia; 23 Psychiatric Genetics, QIMR Berghofer Medical Research Institute, Brisbane, QLD 4006 Australia; 24 Institute of Health and Biomedical Innovation and School of Biomedical Science, Queensland University of Technology, Brisbane, QLD 4059 Australia; 25 23andMe, Mountain View, CA 94041 USA; 26 Department of Obstetrics, Gynecology, and Reproductive Biology, College of Human Medicine, Michigan State University, Grand Rapids, MI 49503 USA; 27 Department of Pathology, Brigham and Women’s Hospital, Harvard Medical School, Boston, MA 02115 USA; 28 Broad Institute of MIT and Harvard, Cambridge, MA 02142 USA; 29 Manchester Centre for Audiology and Deafness, Manchester Academic Health Science Center, University of Manchester, Manchester, M13 9PL UK

**Keywords:** Genome-wide association studies, Reproductive biology, Epidemiology

## Abstract

Uterine leiomyomata (UL) are the most common neoplasms of the female reproductive tract and primary cause for hysterectomy, leading to considerable morbidity and high economic burden. Here we conduct a GWAS meta-analysis in 35,474 cases and 267,505 female controls of European ancestry, identifying eight novel genome-wide significant (*P* < 5 × 10^−8^) loci, in addition to confirming 21 previously reported loci, including multiple independent signals at 10 loci. Phenotypic stratification of UL by heavy menstrual bleeding in 3409 cases and 199,171 female controls reveals genome-wide significant associations at three of the 29 UL loci: 5p15.33 (*TERT*), 5q35.2 (*FGFR4*) and 11q22.3 (*ATM*). Four loci identified in the meta-analysis are also associated with endometriosis risk; an epidemiological meta-analysis across 402,868 women suggests at least a doubling of risk for UL diagnosis among those with a history of endometriosis. These findings increase our understanding of genetic contribution and biology underlying UL development, and suggest overlapping genetic origins with endometriosis.

## Abstract (web summary)

Uterine leiomyomata (UL) or fibroids are neoplasms of the uterine smooth muscle associated with heavy menstrual bleeding and other female reproductive tract morbidity. Here, the authors identify eight previously undescribed genetic loci for UL and further look into genetic overlap with heavy menstrual bleeding and endometriosis.

## Introduction

Uterine leiomyomata (UL), also known as uterine fibroids, are hormone-driven tumors with an estimated prevalence ranging from 20–77%^1,2^. Although the majority of UL are asymptomatic, about 25% of women with UL are symptomatic, and may experience heavy menstrual bleeding (HMB), abdominal pain, infertility, and increased risk of miscarriage^3^. Currently, the only essentially curative treatment is uterine extirpation via total hysterectomy. Known risk factors for UL include increasing age up to menopause, ethnicity (particularly African ancestry), family history of UL, nulliparity, and increased body mass index (BMI)^4^. Studies of familial aggregation and twins, as well as racial differences in prevalence and morbidity, suggest heritable factors influence risk for developing UL^5–10^. Recent GWAS have identified 26 loci significantly associated (*P* *<* 5 × 10^−8^) with UL: 10q24.33, 11p15.5, and 22q13.1 in Japanese women^11^, 25 loci in white women of European ancestry^12–15^, including the three previously identified loci in Japanese women, and a distinct region at 22q13.1 in African American women^16^.

To define further the genetic architecture of UL, we perform a discovery meta-analysis of GWAS on UL across a total of 35,474 cases and 267,505 female controls of white European ancestry, which more than doubles the case sample size of previously reported GWAS^11,12,14–16^. The meta-analysis identifies eight novel loci significantly associated with UL (*P* *<* 5 × 10^−8^) and confirms 21 previously reported European risk loci. Interestingly, HMB-limited UL GWAS reveals three of the 29 independent loci to be significantly associated with the co-occurrence of UL and HMB. Four loci identified in the meta-analysis are also reported to be associated with risk for endometriosis, which together with an epidemiological meta-analysis indicating an association between endometriosis and diagnosis of UL suggest overlapping genetic origins between the two highly common gynecologic diseases.

## Results

### UL GWAS meta-analysis

Our discovery meta-analysis of GWAS on UL includes four population-based cohorts and one direct-to-consumer cohort of white European ancestry: Women’s Genome Health Study (WGHS), Northern Finnish Birth Cohort (NFBC), QIMR Berghofer Medical Research Institute (QIMR), UK Biobank (UKBB), and 23andMe (Supplementary Methods, Supplementary Table 1). Imputation of genotypes was carried out using 1000 Genomes Project Phase 3 and Haplotype Reference Consortium (HRC) reference panels. UL phenotype in each cohort was analyzed in a logistic regression or linear mixed model assuming additive genetic effects with multivariate adjustment for age, BMI, and/or correction for population structure. After quality control metrics were applied, including exclusion of non-informative (MAF < 0.01) and poorly imputed (*r*^2^ < 0.4) SNPs, we performed a fixed-effects, inverse-variance-weighted (IVW) meta-analysis across 35,474 cases with a clinical or self-reported history of UL and 267,505 unaffected female controls. Altogether 8,662,096 biallelic SNPs were analyzed and adjustments for genomic inflation performed (Supplementary Fig. 1, Supplementary Table 2). Through linkage disequilibrium score (LDSC) regression analysis, an estimated 89.5% of the genomic inflation factor (*λ*_GC_) of 1.12 was attributable to polygenic heritability (intercept = 1.02, s.e. = 0.0081). Overall, individual SNP-based heritability (*h*^*2*^) was estimated to be 0.0281 (s.e. = 0.0029) on the liability scale.

### Risk loci associated with UL

**Fig. 1:**
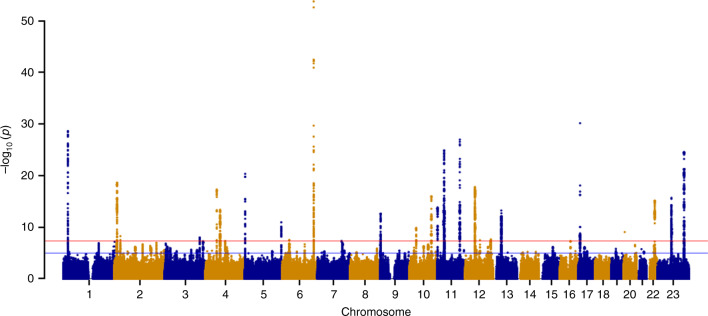
Manhattan plot for UL GWAS meta-analysis across all cohorts. Meta-analysis of GWAS including 302,979 women of white European ancestry across all cohorts identified 29 independent loci associated with UL. Red and blue horizontal lines indicate genome-wide significant (*P* *<* 5 × 10^−8^) and suggestive (*P* *<* 1 × 10^−5^*)* thresholds, respectively

We observe genome-wide significant associations (*P* *<* 5 × 10^−8^) at 2505 SNPs across 29 independent loci (Table 1, Supplementary Fig. 2, Supplementary Table 3). The Manhattan plot is shown in Fig. 1. We identify eight novel loci associated with UL (2p23.2, 4q22.3, 6p21.31, 7q31.2, 10p11.22, 11p14.1, 12q15, and 12q24.31), which include the following candidate genes of interest: *HMGA1*, *BABAM2*, and *WNT2*. *HMGA1* is a member of the high mobility group proteins and is involved in regulation of gene transcription^17^. Somatic rearrangements of *HMGA1* at 6p21 have been recurrently documented in UL, albeit at a much lower frequency than those of *HMGA2*—another member of the high mobility group protein family^18–20^. *BABAM2* at 2p23.2 encodes a death receptor-associating intracellular protein that promotes tumor growth by suppressing apoptosis^21^. Associations at 7q31.2 containing *WNT2*, a member of the Wnt gene family, provide support for the previously suggested role of Wnt signaling in UL^22,23^.

**Table 1:** Overview of lead SNPs with significant associations at 29 independent loci in UL GWAS meta-analysis

Locus	Lead SNP	RA	OA	RAF_EUR_	*P*_Meta_	OR (95% CI)	Gene(s) of interest^a^
1p36.12^b,c^	rs7412010	C	G	0.15	2.4 × 10^−29^	1.13 (1.11–1.16)	*WNT4, CDC42*
2p23.2	rs55819434	A	G	0.91	5.6 × 10^−09^	0.92 (0.90-0.95)	*BABAM2*
2p25.1^b,c^	rs35417544	T	C	0.69	2.3 × 10^−19^	1.09 (1.07–1.10)	*GREB1*
3q26.2^c^	rs35446936	A	G	0.24	1.0 × 10^−08^	0.95 (0.93-0.96)	*TERC*
4q12^c^	rs62323682	T	C	0.94	4.9 × 10^−18^	0.87 (0.84-0.90)	*LNX1, PDGFRA*
4q13.3^c^	rs12640488	A	G	0.52	4.0 × 10^−14^	0.94 (0.92-0.96)	*SULT1B1*
4q22.3	rs4699299	T	C	0.69	4.7 × 10^−08^	0.95 (0.94-0.97)	*PDLIM5*
5p15.33^c^	rs72709458	T	C	0.23	4.7 × 10^−21^	1.10 (1.08–1.13)	*TERT*
5q35.2^c^	rs2456181	C	G	0.49	1.1 × 10^-11^	0.94 (0.93-0.96)	*ZNF346, UIMC1*
6p21.31	rs116251328	A	T	0.02	3.0 × 10^−08^	1.15 (1.09–1.21)	*GRM4, HMGA1*
6q25.2^b,c^	rs58415480	C	G	0.84	1.9 × 10^−54^	0.84 (0.82-0.86)	*SYNE1, ESR1*
7q31.2	rs2270206	A	C	0.16	4.6 × 10^−08^	1.06 (1.04–1.09)	*WNT2*
9p24.3^c^	rs10976689	A	G	0.60	2.4 × 10^-13^	0.94 (0.93-0.96)	*ANKRD15*
10q24.3^c^	rs9419958	T	C	0.13	1.1 × 10^−16^	1.10 (1.08–1.13)	*OBFC1, SLK*
10p11.22	rs10508765	A	G	0.80	1.5 × 10^−10^	1.07 (1.05–1.09)	*ZEB1, ARHGAP12*
11p15.5^c^	rs547025	T	C	0.92	1.5 × 10^−14^	1.13 (1.09–1.16)	*RIC8A, BET1L*
11p14.1^b^	rs11031006	A	G	0.14	5.7 × 10^−15^	0.91 (0.89-0.93)	*FSHB*
11p13^c^	rs61889186	C	G	0.86	1.4 × 10^-25^	0.89 (0.87-0.91)	*WT1*
11p13^c^	rs2785202	C	G	0.55	6.9 × 10^−14^	1.06 (1.05–1.08)	*PDHX, CD44*
11q22.3^c^	rs149934734	T	C	0.03	1.1 × 10^−27^	1.33 (1.26–1.40)	*C11orf65, KDELC2*
12q13.11^c^	rs2131371	A	C	0.28	1.6 × 10^−18^	0.93 (0.91-0.94)	*SLC38A2*
12q15	rs11178393	T	C	0.89	3.3 × 10^−08^	1.08 (1.05–1.10)	*PTPRR*
12q24.31	rs28583837	A	G	0.22	2.3 × 10^−08^	0.94 (0.92-0.96)	*PITPNM2*
13q14.11^c^	rs117245733	A	G	0.02	5.7 × 10^−14^	1.31 (1.21–1.39)	*FOXO1*
17p13.1^c^	rs78378222	T	G	0.99	7.1 × 10^−31^	0.65 (0.60-0.70)	*SHBG, TP53*
20p12.3^c^	rs16991615	A	G	0.07	8.8 × 10^−10^	1.11 (1.07–1.14)	*MCM8, TRMT6*
22q13.1^c^	rs4821939	A	T	0.20	7.8 × 10^−16^	1.08 (1.06–1.10)	*TNRC6B*
Xp26.2^c^	rs12392108	A	T	0.31	5.9 × 10^−46^	1.13 (1.11–1.15)	*RAP2C*
Xq13.1^c^	rs4360450	A	G	0.37	2.1 × 10^−18^	1.08 (1.06–1.10)	*MED12*

*SNP* single-nucleotide polymorphism, *RA* risk allele, *OA* other allele, *RAF*_*EUR*_ average risk allele frequency in European samples, *OR* odds ratio

^a^≤300 kb distant from association signal

^b^Loci previously associated with endometriosis

^c^Loci previously associated with UL

Among 29 independent loci are 21 loci previously reported to be significantly associated with UL^11–16^. A number of identified loci harbor genes previously implicated in cell growth and cancer risk in different tissue types, including cervical cancer^24^, epithelial ovarian cancer^25,26^, breast cancer^27,28^, glioma^29,30^, bladder cancer^31^, and pancreatic cancer^32–34^. Specifically, seven independent loci contain well-characterized oncogenes and tumor suppressor genes from the Cancer Gene Census list in COSMIC^35^: *PDGFRA*, *TERT, ESR1*, *WT1*, *ATM*, *FOXO1*, and *TP53*.

**Fig. 2:**
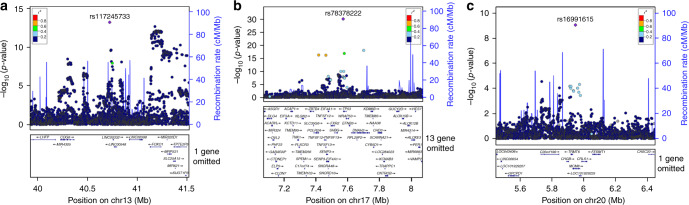
Fine-mapping reveals three association signals with a single driver in 99% credible set. Association with UL is expressed as −log_10_(*P* value) for the three signals on chromosomes: (**a**) 13q14.11, (**b**) 17p13.1, and (**c**) 20p12.3. The labeled SNP represents the most significant SNP for each locus. SNP association *P*-value is shown on the y axis, while SNP position (with gene annotation) appears on the *x* axis. Each SNP is colored according to the strength of LD with the lead SNP. Regional association plots were produced in LocusZoom

Using approximate conditional analysis, we identify multiple distinct association signals for UL at 10 loci (at locus-wide significance, *P* *<* 1 × 10^−5^, Bonferroni correction) (Supplementary Table 4). Fine-mapping was conducted on all 43 distinct association signals arising from the 29 detected UL loci, revealing three association signals with a single variant in the 99% credible set (Fig. 2, Supplementary Table 5). The missense variant at 20p12.3 (rs16991615; E341K) maps to *MCM8*, a gene that encodes a protein involved in DNA double-strand break repair^36^. *MCM8* has also been implicated in length of reproductive lifespan, menopause, and premature ovarian failure^37,38^. Another variant (rs78378222) resides in the 3’UTR of *TP53* at 17p13.1, and has been shown to disturb 3’-end processing of *TP53* mRNA^39^. This variant has been associated with both malignant and benign tumor types^39–41^.

### UL GWAS limited by HMB

**Fig. 3:**
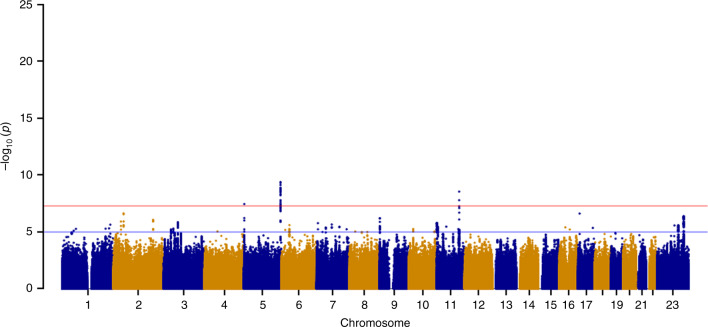
Manhattan plot for GWAS on UL limited by heavy menstrual bleeding. GWAS across 202,580 women of white European ancestry identified three independent loci associated with UL limited by heavy menstrual bleeding. Red and blue horizontal lines indicate genome-wide significant (*P* *<* 5 × 10^−8^) and suggestive (*P* *<* 1 × 10^−5^) thresholds, respectively

HMB, one of the major symptoms of UL, is estimated to affect up to 30% of reproductive-aged women, having a considerable impact on a woman’s quality of life. Thus, variants specifically associated with this symptom are of particular interest for drug target development. We performed a GWAS on UL limited by HMB using a linear mixed model across 3409 cases and 199,171 unaffected female controls from the UKBB (Supplementary Methods, Supplementary Fig. 3). We observe genome-wide significant associations (*P* *<* 5 × 10^−8^) at three of the 29 independent UL loci: 5p15.33 (rs72709458, OR [95% CI] = 0.86 [0.81–0.91], *P* = 3.50 × 10^−8^), 5q35.2 (rs2456181, OR [95% CI] = 0.87 [0.83–0.91], *P* = 4.20 × 10^−10^), and 11q22.3 (rs1800057, OR [95% CI] = 0.66 [0.58–0.76], *P* = 2.80 x 10^−9^) (Fig. 3, Supplementary Fig. 4, Supplementary Table 6). The lead SNP at 11q22.3, a missense variant in *ATM*, has been associated with increased risk of various cancers, such as breast cancer^42,43^, while the lead SNP at 5p15.33, an intronic *TERT* variant, has previously been implicated in gliomas^44^. The lead SNP rs2456181 at 5q35.2 resides near *FGFR4*, a gene encoding a cell-surface receptor for fibroblast growth factors involved in regulation of several pathways, including cell proliferation, differentiation, and migration.

### HMB GWAS

A GWAS based solely on HMB across 9813 cases and 210,946 female controls reveals one genome-wide significant association at 11p14.1, one of the eight novel loci associated with UL (Supplementary Figs. 5 and 6). The lead SNPs for UL and HMB at 11p14.1 are in high LD, and the direction of the effect is the same (Supplementary Fig. 7). This locus has previously been associated with endometriosis, age at menarche, and follicle-stimulating/luteinizing hormone levels^45–47^. According to GTEx (v7), the lead SNP for HMB (rs11031005) is a potential expression quantitative trait locus (eQTL) for *ARL14EP* in several tissue types, such as testis and thyroid. Mendelian randomization (MR) was used to assess the causality of genetic association between UL (exposure) and HMB (outcome). Interestingly, MR reveals that genetic predisposition to UL is causally linked to an increased risk of HMB, with the *β* estimate of 0.26 being significant in the IVW model (*P* = 1.2 × 10^−12^) in the absence of heterogeneity (*P* = 0.13) (Supplementary Table 7). The MR Egger regression shows no significant directional pleiotropy (intercept = 0.01, *P* = 0.36) supporting a causal relationship.

### Overlap of UL and endometriosis

Interestingly, significant association signals are observed at several loci previously associated with endometriosis: 1p36.12 (rs7412010, OR [95% CI] = 1.13 [1.11–1.16], *P* = 2.43 × 10^−29^), 2p25.1 (rs35417544, OR [95% CI] = 1.09 [1.07–1.10], *P* = 2.32 × 10^−19^), 6q25.2 (rs58415480, OR [95% CI] *=* 1.19 [1.17–1.22], *P* = 1.86 × 10^−54^), and 11p14.1 (rs11031006, OR [95% CI] = 1.10 [1.07–1.12], *P* = 5.65 × 10^−15^)^45,48–50^. LD is strong between UL and previously reported endometriosis lead SNPs^45^ at all except one locus, 2p25.1 (Supplementary Table 8). In addition, the direction of effect is the same between the lead SNPs at 1p36.12. Using LDSC regression, we observe a moderate genetic correlation between UL and endometriosis in women with European ancestry (*r*_*g* = _0.39, s.e = 0.05, *P* = 9.77 × 10^−13^). Endometriosis has an earlier age-of-onset than UL, with a mean age of 25–29 years and 35 years, respectively. MR suggests that genetic predisposition to endometriosis (exposure) is causally linked to an increased risk of UL (outcome); the *β* of 0.36 is significant (*P* = 3.7 × 10^−3^) in the IVW model (heterogeneity *P* = 9.5 × 10^−68^) (Supplementary Table 7). Leave-one-out sensitivity analysis reveals that no single SNP alone drives the significant relationship between endometriosis and UL, but instead the relationship is accounted for by contributions from multiple variants across the genome (Supplementary Fig. 8). Given the high degree of heterogeneity, the effect sizes were estimated in a minimal set of SNPs that when used as a genetic instrument eliminate the heterogeneity (Supplementary Fig. 9). The effect size estimate (*β* = 0.12) from the minimal set of variants remains significant (*P* = 4.3 × 10^−3^) in the IVW model in the absence of heterogeneity (*P* = 0.23). We also applied the MR pleiotropy residual sum and outlier (MR-PRESSO) global and distortion tests to adjust for variants causing significant bias in the estimates through horizontal pleiotropy. Outlier-adjusted estimates still provide significant evidence for a causal estimate of endometriosis on UL (*β* *=* 0.29, *P* = 0.002) (Supplementary Table 7).

Endometriosis, defined by ectopic growth of endometrial-like tissue outside the uterus, is a common inflammatory hormone-dependent disease that affects reproductive-aged women^51^. Although functional studies of relevant tissue are needed to confirm consequences of the variants in regulation of gene expression, each of the four observed overlapping genomic loci contains a gene(s) known to be involved in progesterone or estrogen signaling. *WNT4* at 1p36.12 encodes a secreted signaling factor that promotes female sex development, and regulates both postnatal uterine development and progesterone signaling during decidualization^52,53^. Recently, SNPs at 1p36.12 associated with a greater endometriosis risk have been suggested to act through *CDC42*, a gene that encodes a small GTPase of the Rho family^54^. *GREB1* at 2p25.1 is an early response gene in the estrogen receptor (ER)-regulated pathway, and promotes growth of breast and pancreatic cancer cells^55,56^. *ESR1* at 6q25.2 encodes the alpha subunit of the ligand-activated nuclear ER that regulates cell proliferation in the uterus^57^. *FSHB* at 11p14.1 encodes the biologically active subunit of follicle-stimulating hormone, which regulates maturation of ovarian follicles and release of ova during menstruation^58,59^.

### Epidemiological meta-analysis

**Fig. 4:**
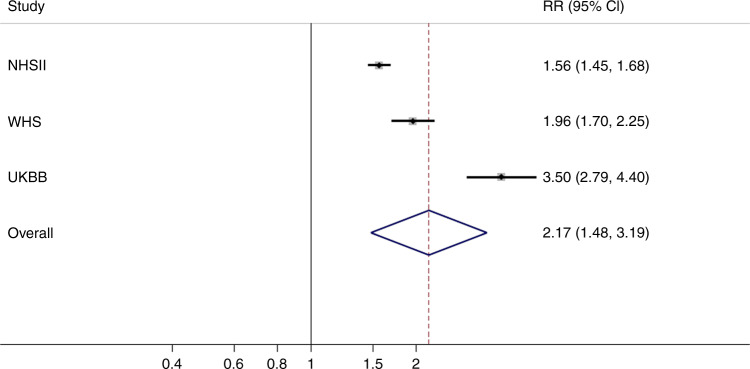
Epidemiologic meta-analysis demonstrates endometriosis is associated with UL. Random-effects, inverse-variance-weighted meta-analysis was performed across the effect sizes and standard errors in 402,868 women from three cohorts (NHSII, WHS, and UKBB). Squares represent point estimates from individual studies, whiskers correspond to the 95% CIs, and the diamond represents results from the meta-analysis. There was evidence of significant heterogeneity based on Cochran’s Q statistic (*P* *<* 1 × 10^−4^)

Given shared risk loci and genetic correlation of UL and endometriosis, we conducted an epidemiological meta-analysis including 402,868 women from three population-based cohorts: Nurses’ Health Study II (NHSII), Women’s Health Study (WHS), and UKBB (Supplementary Methods, Supplementary Table 9), to assess the likelihood of UL diagnosis among women who had or had not been diagnosed with endometriosis. Women with endometriosis had a significantly higher likelihood of UL diagnosis (multivariable-adjusted summary relative risk (RR) [95% CI] = 2.17 [1.48–3.19]) (Fig. 4). All cohort-specific analyses demonstrated at least a doubling of risk, suggesting a robust association (Table 2). However, biologically and statistically significant heterogeneity was observed in the pooling of effect size estimates in the meta-analysis (*P* < 1 × 10^−4^) (Fig. 4). Therefore, absolute effect estimates need to be interpreted in the context of source populations. Heterogeneity could reflect various different population sampling and data collection factors among the three cohorts. First, the validity of self-reported diagnosis of endometriosis and to a lesser extent UL are known to be <75% in general population cohorts, such as UKBB, compared to more highly validated self-assessment in the medical professional NHSII and WHS cohorts^7^. Second, endometriosis clinical definitions prior to the 1990s were more restrictive—often limited to the presence of endometrioma and/or “powderburn” superficial peritoneal lesions among adult women^60^. Subsequently, definitions have expanded to recognize a wide range of superficial peritoneal phenotypic presentations, as well as incidence among adolescents and young women^61^. It may be impactful therefore that the WHS participants were ≥ 45 years of age in 1992, while NHSII participants were ≥ 25 years of age in 1989, and UKBB participants were aged 40 to 69 in 2006. Thus, disease definitions varied during the peak calendar years of incidence among the cohorts, and in addition, while the NHSII were queried about endometriosis prospectively during their reproductive years, the WHS and UKBB cohorts were cross-sectionally asked to recall their gynecologic health experience decades earlier. It is also important to note that while WHS and NHSII participants were asked specifically about endometriosis diagnosis via questionnaire, the UKBB data collection included qualitative interviews during which endometriosis would be documented only when the participant herself raised it as a health issue. Those with mild symptoms or those past their reproductive years and thus past the moderate to severe life-impacting symptoms of the disease may have been less likely to offer endometriosis among the list of their health issues. This is supported by the low prevalence of endometriosis reported within the UKBB compared to WHS, NHSII, and other population-based estimates^62^. However, the UKBB participants (due to the qualitative interview structure and recall bias) and the WHS participants (due to recall bias) could have been more likely to choose to report endometriosis if they also suffered from UL together, resulting in diagnostic bias and consequently an inflation of effect estimates. Indeed, the population heterogeneity and differing potential for diagnostic bias by cohort fits with the observed differences among effect estimate magnitudes with the RRs and CI widths ordered from NHSII (RR = 1.56) to WHS (RR = 1.96) to UKBB (RR = 3.50) (Fig. 4).

**Table 2:** Multivariable-adjusted effect estimates of the association between endometriosis and UL among women in NHSII, WHS, and UKBB cohorts

Cohort	*n*	UL cases	Age-adjusted	Multivariable-adjusted
Nurses’ Health Study II^a^	102,545	10,714	1.61 (1.50–1.73)	1.57 (1.45–1.68)
Women’s Health Study^b^	26,868	1,262	2.04 (1.78–2.34)	1.97 (1.71–2.26)
UK Biobank^c^	273,455	19,789	3.11 (2.86–3.37)	3.50 (2.77–4.38)

^a^Hazard ratios (95% confidence intervals [CIs]) from Cox regression models. Multivariable model was adjusted for age (continuous), age at menarche (<11, 11, 12, 13, 14–15, >15), infertility (yes, no), ancestry (White, Black, Hispanic, Asian, other), parity (nulliparous, 1, 2, 3, 4+), age at first birth (<25, 25–29, >29), time since last birth (<1, 1–3, 4–5, 6–7, 8–9, 10–12, 13–15, ≥16), age first oral contraceptive use (13–16, 17–20, 21–24, ≥25), BMI (<20, 20–21.9, 22–23.9, 24–24.9, 25–26.9, 27–29.9, ≥30), menstrual cycle length (<26, 26–31, 32–50, and >50 days), smoking (never, past, current), recent gynecologic/breast exam (no recent exam, recent exam), use of anti-hypertensive medications/diastolic blood pressure (no meds <65, no meds 65–74, no meds 75–84, no meds 85–89, no meds ≥90, meds <65, meds 65–74, meds 75–84, meds 85-89, meds ≥90), and physical activity (MET hours/week: <3, 3–<9, 9–<18, 18–<27, 27–<42, ≥42).

^b^Odds ratios (95% CI) from logistic regression models. Multivariable model was adjusted for age at baseline (continuous), age at menarche (<11, 11, 12, 13, 14–15, >15), ancestry (White, Black, Hispanic, Asian, other), parity (nulliparous, 1, 2, 3, ≥4), age at first birth (<25, 25–29, >29), oral contraceptive use (ever, never), BMI (<20, 20–21.9, 22–23.9, 24–24.9, 25–26.9, 27–29.9, ≥30), smoking (never, past, current), use of anti-hypertensive medications/diastolic blood pressure (no meds <65, no meds 65–74, no meds 75-84, no meds 85–89, no meds ≥90, meds <65, meds 65–74, meds 75–84, meds 85–89, meds ≥ 90), physical activity (never/rarely, <1 time/week, 1–3 times.week, ≥4 times/week), alcohol consumption (never/rarely, 1–3 drinks/month, 1–6 drinks/week, ≥1 drinks/day).

^c^Odds ratios (95% CI) from logistic regression models. Multivariable model was adjusted for age (<50, 50–55, 56–60, ≥60), age at menarche (<11, 11, 12, 13, 14–15, >15), ancestry (White, Black, Asian, other), parity (nulliparous, 1, 2, 3, ≥4), age at first birth (<25, 25–29, >29), oral contraceptive use (ever, never), BMI (<20, 20–21.9, 22–23.9, 24–24.9, 25–26.9, 27–29.9, ≥30), smoking (never, past, current), physical activity (never/rarely, 1–3 drinks/week, ≥4 drinks/week), alcohol consumption (never/rarely,1–3/month, 1–4/week, ≥1/day), and menopausal status (premenopausal, postmenopausal).

### Bioinformatic analyses of UL risk SNPs and loci

To estimate the genetic correlation between UL and various reproductive traits, as well as cardiometabolic traits/diseases, we performed LD Hub analysis for a total of 21 traits/diseases (Supplementary Data 1). We observe significant correlations between increased risk of UL and earlier age of menarche (*r*_*g*_ = −0.16, *P* = 3.7 × 10^−6^), earlier age of first birth (*r*_*g*_ = −0.14, *P* = 1.0 × 10^−3^), increased levels of triglycerides (*r*_*g*_ = 0.13, *P* = 1.9 × 10^−3^), and increased BMI (*r*_*g*_ = 0.11, *P* = 2.0 × 10^−3^), as previously suggested by epidemiological studies^63,64^, illustrating that common genetic factors can predispose women to both risk factors related to, for example, adverse metabolic and cardiovascular disease risk and UL. Gene-set and tissue enrichment analyses across 8971 SNPs with suggestive (*P* *<* 1 × 10^−5^) or significant (*P* *<* 5 × 10^−8^) UL associations using DEPICT^65^ reveal enrichments (false discovery rate (FDR) < 0.05) in gene sets, such as steroid hormone receptor (GO:0035258; *P* = 1.03 × 10^−5^), hormone receptor binding (GO:0051427; *P* = 9.07 × 10^−5^), and nuclear hormone receptor binding (GO:0035257; *P* = 1.53 × 10^−4^) (Supplementary Data 2 and 3). The results are concordant with the hormone-driven nature of UL. We did not observe any cell/tissue types significantly enriched for the expression of the genes in the associated loci (Supplementary Fig. 10). To identify potential causal genes at UL risk loci, we used a summary-data based MR (SMR) method, including both eQTL and mQTL data from peripheral blood^66,67^. We identify 18 potential causal genes showing no significant heterogeneity in SMR (*P*_HEIDI_ > 5 × 10^−3^), including *WNT4* (rs55938609, *P*_SMR_ = 6.92 × 10^−15^), *GREB1* (rs35417544, *P*_SMR_ = 3.93 × 10^−19^), *WT1* (rs12280757, *P*_SMR_ = 1.87 × 10^−18^), and *FOXO1* (rs3924478, *P*_SMR_ = 5.76 × 10^−10^) (Supplementary Data 4 and 5).

### FOXO1 expression in UL

To explore potential functional significance, we examined expression of the FOXO1 protein, a transcription factor that plays an important role in cell proliferation, apoptosis, DNA repair, and stress response^68^. Interestingly, inactivation of *FOXO1* promotes cell proliferation and tumorigenesis in several hormone-regulated malignancies, such as prostate, breast, cervical, and endometrial cancers^69–72^. Conversely, we observe a significant increase in nuclear FOXO1 protein expression in UL compared to myometrial samples using immunohistochemistry on tissue microarrays (Supplementary Fig. 11). Patient-matched tumor-normal pairs show 1.69-fold higher (*P* = 0.01; paired *t*-test) nuclear FOXO1 expression in UL, while the expression is as much as 2.32-fold greater (*P* *=* 1.52 × 10^−9^; Welch’s *t*-test) when all 335 UL are considered (Supplementary Fig. 12). These results are consistent with a previous study^73^, which showed phosphorylated (p) FOXO1 (pSer^256^) to be predominantly present in the nucleus in UL, but sequestered in the cytoplasm of myometrium. The concomitant increase of p-FOXO1 and reduced expression of its interaction partner 14-3-3${\mathrm{\gamma }}$ in UL has been suggested to lead to impaired nuclear/cytoplasmic shuttling of p-FOXO1, which promotes cell survival^73–75^. We performed stratification of samples by genotype, revealing a statistically significant increase in FOXO1 levels of UL harboring the risk allele for rs6563799 (allelic dosage, *P* = 0.047; homozygosity for risk allele, *P* = 0.035) (Supplementary Figs. 11 and 13). An increase in FOXO1 levels of UL with the rs7986407 risk allele is also observed; however, the change is not statistically significant (Supplementary Figs. 11 and 13).

## Discussion

In our meta-analysis of GWAS on UL, we identify 29 genomic loci to be significantly associated with UL in women of white European ancestry, including eight novel and 21 previously reported loci. Candidate genes in the identified loci implicate pathways of estrogen and progesterone signaling (*ESR1*, *FSHB, GREB1, WNT2*, and *WNT4*), as well as cell growth (*FOXO1*, *PDGFRA, TERT*, *TERC*, and *TP53*) in predisposing women to UL. We do not confirm five of 26 previously identified loci reported to be significantly associated with UL^12,14–16^. Two of these loci, 3p24.1 and 16q12.1, are nominally significant (*P* *<* 1 × 10^−5^) in our GWAS meta-analysis, but the remaining three loci (3q29, 17q25.3 and a distinct region at 22q13.1) do not reach nominal significance. Ancestral differences may explain the absence of the association originally identified in African American women in the genomic region at 22q13.1, while variation in phenotypic definitions^12^ may underlie the two other loci.

Discovery of eight novel loci significantly associated with UL reveals several candidate genes of particular interest: *BABAM2, FSHB, HMGA1*, and *WNT2*. Because UL are benign tumors that rarely, if ever, develop into malignancy, the association between UL and multiple loci harboring well-known oncogenes and tumor suppressor genes is also worthy of note. Fine-mapping of the *TP53* locus identifies rs78378222 to be the most probable causal variant, which has been shown to disrupt the polyadenylation sequence in the 3’UTR of *TP53* and result in reduced expression of mRNA^39^. We also observe nuclear FOXO1 levels to be significantly elevated in UL when compared to myometrium. FOXO1 is a downstream target of the Akt signaling pathway that responds to hormone signaling through the progesterone receptor in UL and activates proliferative responses^76^.

HMB is one of the major debilitating symptoms of UL and can have a substantial impact on a woman’s quality of life. Here, we report GWAS on both UL limited by HMB and solely on HMB, revealing potential targets for pharmacologic intervention: *ARL14EP*, *ATM*, *TERT*, and *FGFR4*. In addition, MR analyses suggest that genetic predisposition to UL is causally linked to an increased risk of HMB. These results form a solid basis for further work to elucidate the mechanisms underlying UL-related HMB and towards tailored treatments of UL and HMB.

Biological overlap between UL and endometriosis, two highly common gynecologic diseases has long been suspected due to similarities in molecular mechanisms and progenitor cells. Our UL GWAS meta-analysis indicates that genes previously associated with endometriosis and involved in hormone-signaling pathways are also associated with UL (*WNT4/CDC42*, *GREB1*, *ESR1*, and *FSHB*). Overlap observed in the genetic etiology of endometriosis and UL led us to epidemiologically quantify the co-occurrence of these two diseases across three independent cohorts. The epidemiological meta-analysis indicates that women with a history of endometriosis are at elevated risk for reporting UL. Results from our MR analyses suggest that genetic predisposition to endometriosis is causally linked to increased risk of UL. Alternatively, given the discordance in the direction of allelic effects for the UL and endometriosis loci, our MR results may indicate a significant overlap in the underlying biology of the two diseases. Additional work is needed to better quantify the contribution of genetic effects to the directional relationship between endometriosis and UL. Results of which will enable us to quantify what portion of the MR results reflect the fundamental pathobiological overlap in these two diseases of the uterus. Further characterization of the mutual pathogenic mechanisms of UL and endometriosis has the capacity to discover not only a deeper understanding of the underlying biology, but also treatments for two diseases that cause significant morbidity in roughly one-third of the world’s population.

## Methods

### Subjects

For UL GWAS meta-analysis, four population-based cohorts (WGHS, NFBC, QIMR and UKBB) and one direct-to-consumer cohort (23andMe) from the FibroGENE consortium were included (Supplementary Table 1), resulting in 35,474 UL cases and 267,505 female controls of white European ancestry. Sample sizes were maximized using a basic, harmonizing phenotype definition to separate cases and controls solely based on either self-report or clinically documented UL history. Our large-scale epidemiologic analysis was comprised of three population-based cohorts (NHSII, WHS, and UKBB), totaling 402,869 women. HMB GWAS included the UKBB cohort, consisting of 220,759 women. Detailed descriptions of cohorts and sample selections are available in Supplementary Methods. All participants provided informed consent in accordance with the processes approved by the relevant jurisdiction for human subject research for each cohort: the Partners HealthCare System Human Research Committee (WHS/WGHS), the Ethical Committee of the Northern Ostrobothnia Hospital District (NFBC), the Human Research Ethics Committee at the QIMR Berghofer Medical Research Institute and the Australian Twin Registry (QIMR), the North West Multi-centre Research Ethics Committee (UKBB), Ethical and Independent Review Services (an external institutional review board; 23andMe), and the Institutional Review Boards at Harvard T.H. Chan School of Public Health and Brigham and Women’s Hospital (Partners Human Research Committee) (NHSII).

### Genotyping

Several different Illumina-based genotyping platforms (Illumina Inc., San Diego, CA, USA) were used: HumanHap300 Duo‘+’ chips or the combination of the Human-Hap300 Duo and iSelect chips (WGHS), Infinium 370cnvDuo array (NFBC), 317 K, 370 K, or 610 K SNP platforms (QIMR). Genotyping of participants in the UKBB was performed either on the Affymetrix UK BiLEVE or Affymetrix UK Biobank Axiom® array with over 95% similarity. Genotyping of participants in the 23andMe cohort was performed on various versions of Illumina-based BeadChips.

### Quality control and imputation

Each cohort conducted quality control measures and imputation for their data. For WGHS, NFBC, QIMR, and 23andMe, all cases and controls with a genotyping call rate <0.98 were excluded from the study. Imputation was performed on both autosomal and sex chromosomes using the reference panel from the 1000 Genomes Project European dataset (1000 G EUR) Phase 3. Imputation was carried out using ShapeIt2 and IMPUTE2 softwares^77,78^. SNPs with call rates of <99% or with deviation from Hardy-Weinberg equilibrium (*P* ≤ 1 × 10^−6^) were excluded from further analyses. Population stratification for the data was examined with principal component analysis (PCA) using EIGENSTRAT^79^. The four HapMap populations were used as reference groups: Europeans (CEU), Africans (YRI), Japanese (JPT), and Chinese (CHB). All observed outliers were removed from the study. UKBB data QC and imputation were performed centrally, prior to public release of the data^80^. Genotype data used in the present analyses were imputed up to the Haplotype Reference Consortium (HRC) panel. We applied additional quality control filters to exclude poorly imputed SNPs (*r*^2^ < 0.4) and SNPs with a MAF of <1%.

### Association analyses

Using additive encoding of genotypes and adjusting for age, BMI, and/or the first five PCs, logistic regression analysis was performed in WGHS, NFBC, QIMR, and 23andMe cohorts and summary statistics were provided, including beta coefficients, *χ*^2^ values, and standard errors, for genotyped and imputed SNPs. The UKBB association analyses were conducted using a linear mixed model (BOLT-LMM v.2.3.2)^81^ adjusting for the two array types used, age and BMI (fixed effects), and a random effect accounting for relatedness between women. Effect size estimates (*β* and SE) from the linear mixed-model were converted to log-odds scale prior to meta-analysis. A fixed-effects, inverse-variance-weighted (IVW) meta-analysis on summary statistics was conducted using METAL^82^ across all cohorts (Supplementary Data 6). A total of 8,662,096 SNPs were available from at least two of the five cohorts. A quantile-quantile plot of the results from meta-analysis across all GWAS cohorts is shown in Supplementary Fig. 1. Details on the overall genomic inflation factor and number of analyzed SNPs for each cohort are provided in Supplementary Table 2. For GWAS meta-analysis, independence of genetic association with UL was defined as SNPs in low LD (*r*^2^ < 0.1) with nearby (≤500 kb) significantly associated SNPs. Individual loci correspond to regions of the genome containing all SNPs in LD (*r*^2^ > 0.6) with index SNPs. Any adjacent regions within 250 kb of one another were combined and classified as a single locus of association. All associated genomic regions were confirmed to have lead SNPs that were either directly genotyped or that met a rigorously high quality imputation threshold (INFO > 0.9) in at least two cohorts.

### Linkage disequilibrium score regression (LDSC)

Analysis of residual inflation in test statistics was conducted using univariate LDSC regression. Individual *χ*2 values for each SNP analyzed in the GWAS meta-analysis was regressed onto LD scores estimated from the 1000 G EUR panel. Heritability calculations can be derived from analyzing the slope and *y*-axis intercept of the slope of the regression line. Percent impact of confounders, such as population stratification, on test statistic inflation are quantified as the LDSC ratio [((intercept–1))/((mean *χ*^2^–1))] × 100%. Remaining effects [(1–LDSC ratio) × 100%] represent the percentage of inflation attributed to polygenic heritability. Univariate LDSC regression was conducted using the LDSC software (https://github.com/bulik/ldsc.git). Adjustment of heritability (*h*^2^) calculations to the liability scale were performed by accounting for the prevalence of UL in the sample (~0.132) compared to the general population (~0.300). LDSC software was also used to estimate the genetic correlation between UL and endometriosis (Endo) using endometriosis GWA meta-analysis summary data from Sapkota et al.^45^ consisting of only European cohorts. The heritability and LD score intercepts for both traits were computed, in this analysis with SNPs present in both datasets for LDSC regression again using LD scores from the 1000 G EUR panel. Genetic correlation between traits was estimated as the genetic covariance among SNPs / √ *h*^*2*^_UL_ × *h*^*2*^_Endo_.

### Approximate conditional analysis

Approximate conditional analysis, implemented in GCTA^83^, was conducted to dissect distinct signals of association at each locus. Of note, where lead SNPs at adjacent loci mapped within 1 Mb of each other, loci were combined as a single region for conditional analysis, to account for potential LD between SNPs in different loci. GCTA makes use of meta-analysis association summary statistics (log-OR and corresponding standard error) and a reference panel of individual-level genotype data to obtain LD between all pairs of SNPs at a locus (or region) that approximates the covariance in effect estimates in a joint model. For these analyses, we made use of 5000 randomly selected white British women (of European descent) as reference. We used the -cojo-slct option to select index variants for each distinct association signal, at a locus-wide significance threshold of *P* < 10^−5^, which is a conservative Bonferroni correction for the number of SNPs mapping to a locus. For loci with multiple distinct association signals, we obtained the conditional association summary statistics for each by conditioning on all other index SNPs at the locus (or region) using the -cojo-cond option.

### Fine-mapping distinct association signals

For each distinct association signal, association summary statistics (log-OR and corresponding standard error) were extracted from the meta-analysis for all SNPs at the locus (or region). For loci with a single signal of association, we made use of association summary statistics from the unconditional meta-analysis. For loci with multiple signals of association, we made use of association summary statistics from the approximate conditional analysis. For each SNP *j*, we calculated an approximate Bayes’ factor in favor of association^84^, given by

1 $$\Lambda _j = \sqrt {\frac{{V_j}}{{V_j + \omega }}} {\mathrm{exp}}\left[ {\frac{{\omega \beta _j^2}}{{2V_j\left( {V_j + \omega } \right)}}} \right],$$

where *β*_*j*_ and *V*_*j*_ denote the estimated log-OR and corresponding variance from the meta-analysis. The parameter *ω* denotes the prior variance in allelic effects, taken here to be 0.04 for a disease outcome^84^. We then calculated the posterior probability, *π*_*j*_, that the *j*th SNP is causal for the association signal, given by

2 $$\pi _{Cj} = \frac{{{\it{\Lambda }}_j}}{{\mathop {\sum}\nolimits_k {{\it{\Lambda }}_k} }},$$

where the summation is over all retained variants in the locus (or region). The 99% credible set for each signal was then constructed by: (i) ranking all variants according to their Bayes’ factor, *Λ*_*j*_; and (ii) including ranked variants until their cumulative posterior probability of causality is at least 0.99.

### Heavy menstrual bleeding (HMB) GWAS

The HMB GWAS was conducted using data from the UKBB cohort (Supplementary Methods). Both hospital-linked medical records and self-report were considered to identify women with a history of UL, while for HMB only hospital-linked medical records were taken into account. Controls had no previous history of either UL or HMB. Association analyses were performed using a linear mixed model (BOLT-LMM v.2.3.2)^81^. Effect size estimates (*β* and SE) from the linear mixed-model were converted to log-odds scale.

### Mendelian randomization (MR)

MR analyses were performed using the Two Sample Mendelian Randomization R package. GWAS summary statistics on HMB from the UKBB cohort were used to create outcome data for MR between UL (exposure) and HMB (outcome). To avoid overlap between samples in the exposure and outcome cohorts, we performed UL GWAS excluding all the HMB cases^85^. LD pruning was performed to confirm no duplication of exposure haplotypes or SNPs. Subsequently, data were harmonized to ensure the same reference alleles were used in exposure and outcome GWAS and that the variants were present in both GWAS datasets. Thirteen independent SNPs associated with UL from our GWAS meta-analysis were available in the HMB GWAS summary data to test for a causal effect of UL on HMB. There were too few significant SNPs available for HMB to test for a causal effect of HMB on UL.

GWAS summary statistics on endometriosis (with laparoscopy, without laparoscopy, and all self-reported endometriosis cases) from the WHS cohort were used to create outcome data for MR between UL (exposure) and endometriosis (outcome). To avoid overlap between samples in the exposure and outcome cohorts, WGHS was excluded from the UL GWAS for MR analysis. Twenty-two independent SNPs associated with UL were available in the endometriosis GWAS summary data to test for a causal effect of UL on endometriosis. For reverse causation model, summary statistics from seven GWAS listing ‘endometriosis’ as the phenotype of interest were available from the EMBL-EBI NHGRI GWAS catalog (Study Accession: GCST000797, GCST001894, GCST001720, GCST005906, GCST000916, GCST004549, GCST004873). Due to a low number of cases/controls or insufficient number of SNPs after LD pruning and data harmonizing, only one of the studies (GCST004549) was included in the analysis. Sixteen independent SNPs associated with endometriosis were available in our UL GWAS summary data to test for a causal effect of endometriosis on UL. The IVW model was used to test causality between exposure and outcome. In addition, the IVW (Q) method was used to test for heterogeneity, leave-one-out sensitivity analysis to identify the effect of individual SNPs, and MR Egger for horizontal pleiotropy. Due to heterogeneity in our initial MR estimates, we have now leveraged a similar approach to the one published in Corbin et al., 2016, to identify the minimum set of variants that when used as a genetic instrument eliminate heterogeneity^86^. We also conducted the MR-PRESSO test to identify and adjust for variants causing significant bias through horizontal pleiotropy^87^. MR-PRESSO method (1) applies a global test to evaluate whether horizontal pleiotropy is present, (2) calculates the causal estimates incorporating correction for the detected horizontal pleiotropy, and (3) applies a distortion test to evaluate if the causal estimate is significantly different after adjustment for outliers. We have reported the initial estimates along with the outlier-adjusted estimates as both the global and distortion tests showed significant results.

### Co-morbidity analyses

Each cohort was analyzed individually with study-specific models chosen and covariates coded as appropriate for each cohort’s data structure (Supplementary Methods). The study-specific effect estimates were combined using meta-analysis to obtain a summary RR. Between study heterogeneity was assessed with Cochran Q statistic and the I^2^ statistic^88^. Because heterogeneity among the studies was identified, we reported a random-effects IVW effect estimate based on the DerSimonian and Laird method^89^.

### LD Hub, gene-set, cell/tissue enrichment, and SMR analyses

LD Hub analysis^90^ was conducted using summary-level results data of UL GWAS meta-analysis to estimate the genetic correlation between UL and 21 different traits/diseases, including various reproductive traits and cardiometabolic traits/diseases that have publicly available GWAS results on the LD Hub repository. Multiple-testing correction was performed (0.05/21 = 2.4 × 10^−3^). For gene-set and cell/tissue enrichment, summary statistics from the set of 8971 SNPs with suggestive (*P* *<* 1 × 10^−5^) or significant associations (*P* *<* 5 × 10^−8^) were analyzed using the Data-driven Expression-Prioritized Integration for Complex Traits (DEPICT) software^65^. Using the 1000 G EUR panel as a reference for LD calculations and the ‘clumping’ algorithm in PLINK^91^, we identified 104 independent loci at the suggestive threshold for DEPICT analyses (Supplementary Data 2). FDR < 0.05 was considered statistically significant. For SMR analysis, SNPs present in at least two studies in the summary statistics were considered. The analysis was run using eQTL data from the CAGE blood dataset^66^ and mQTLs from the LBC_BSGS blood dataset^67^.

### FOXO1 immunohistochemistry and genotyping

FOXO1 immunostaining was performed on two replicate tissue microarrays (TMAs) containing 335 UL and 36 myometrium tissue samples from 200 white women of European ancestry obtained from myomectomies and hysterectomies. Tissue cores on the replicate TMAs represent different regions of the same samples, which include corresponding tumor-normal tissue pairs from 35 women. Immunohistochemistry was carried out using the BOND staining system (Leica Biosystems, Buffalo Grove, IL) with a primary antibody dilution 1:100 (clone C29H4, Cell Signaling Technology, Danvers, MA) and hematoxylin as the counterstain. Immunostaining was analyzed using Aperio ImageScope software (Leica Biosystems). Each core was evaluated for the ratio of stain to counterstain taking into account variable cellularity between cores. Only nuclear labeling of the protein was evaluated. The average stain-to-counterstain ratio was compared between patient-matched UL and myometrium samples using a paired *t*-test (two-tailed), while an unpaired *t*-test (Welch’s *t*-test, two-tailed) was applied to compare all UL and myometrium samples. Genomic DNA from 109 UL on the TMA was available for genotyping. These UL were genotyped for two SNPs with genome-wide significance at the 13q14.11 locus: rs6563799 and rs7986407. For each SNP, the average FOXO1 stain-to-counterstain ratio was compared across increasing dosage of the risk allele using a one-way analysis of variance test (two-tailed). We also performed an unpaired *t*-test to compare mean expression of UL homozygous for the risk variant against the other genotypes (Welch’s *t*-test, two-tailed). *P*-values < 0.05 were considered statistically significant.

### URLs

For WHS see http://whs.bwh.harvard.edu/; for NFBC see http://www.oulu.fi/nfbc/; for QIMR see http://www.qimrberghofer.edu.au/; for UK Biobank see http://www.ukbiobank.ac.uk/; for 23andMe see https://research.23andme.com/; for METAL see http://csg.sph.umich.edu/abecasis/metal/; for LDSC see https://github.com/bulik/ldsc.git; for DEPICT see https://data.broadinstitute.org/mpg/depict/; for SMR see http://cnsgenomics.com/software/smr/; and for PLINK see http://pngu.mgh.harvard.edu/purcell/plink/.

### Reporting summary

Further information on research design is available in the Nature Research Reporting Summary linked to this article.

## Supplementary information

Supplementary Information

Peer Review File

Reporting Summary

Description of Additional Supplementary Files

Supplementary Data 1

Supplementary Data 2

Supplementary Data 3

Supplementary Data 4

Supplementary Data 5

Supplementary Data 6

## Data Availability

The authors declare that the data supporting the findings of this study are available within the article and its Supplementary Information files. Summary statistics for the top 10,000 UL GWAS meta-analysis variants are provided in Supplementary Data 6. UL GWAS meta-analysis summary statistics (without 23andMe), UL GWAS limited by HMB and HMB GWAS summary statistics will be made available through the NHGRI-EBI GWAS Catalog https://www.ebi.ac.uk/gwas/downloads/summary-statistics. To request access to 23andMe GWAS summary statistics, please visit https://research.23andme.com/dataset-access/.
