# Author Correction: Genome-wide association and epidemiological analyses reveal common genetic origins between uterine leiomyomata and endometriosis

**DOI:** 10.1038/s41467-022-33222-y

**Published:** 2022-09-21

**Authors:** C. S. Gallagher, N. Mäkinen, H. R. Harris, N. Rahmioglu, O. Uimari, J. P. Cook, N. Shigesi, T. Ferreira, D. R. Velez-Edwards, T. L. Edwards, S. Mortlock, Z. Ruhioglu, F. Day, C. M. Becker, V. Karhunen, H. Martikainen, M.-R. Järvelin, R. M. Cantor, P. M. Ridker, K. L. Terry, J. E. Buring, S. D. Gordon, S. E. Medland, G. W. Montgomery, D. R. Nyholt, D. A. Hinds, J. Y. Tung, the 23andMe Research Team, Michelle Agee, Babak Alipanahi, Adam Auton, Robert K. Bell, Katarzyna Bryc, Sarah L. Elson, Pierre Fontanillas, Nicholas A. Furlotte, Karen E. Huber, Aaron Kleinman, Nadia K. Litterman, Matthew H. McIntyre, Joanna L. Mountain, Elizabeth S. Noblin, Carrie A. M. Northover, Steven J. Pitts, J. Fah Sathirapongsasuti, Olga V. Sazonova, Janie F. Shelton, Suyash Shringarpure, Chao Tian, Vladimir Vacic, Catherine H. Wilson, J. R. B. Perry, P. A. Lind, J. N. Painter, N. G. Martin, A. P. Morris, D. I. Chasman, S. A. Missmer, K. T. Zondervan, C. C. Morton

**Affiliations:** 1 Department of Genetics, Harvard Medical School, Boston, MA 02115 USA; 2 Department of Obstetrics and Gynecology, Brigham and Women’s Hospital, Harvard Medical School, Boston, MA 02115 USA; 3 Program in Epidemiology, Division of Public Health Sciences, Fred Hutchinson Cancer Research Center, Seattle, WA 98109 USA; 4 Wellcome Centre for Human Genetics, University of Oxford, Oxford, OX3 7BN UK; 5 Endometriosis CaRe Centre, Nuffield Department of Women’s and Reproductive Health, University of Oxford, John Radcliffe Hospital, Oxford, OX3 9DU UK; 6 Department of Obstetrics and Gynecology, Oulu University Hospital and PEDEGO Research Unit & Medical Research Center Oulu, University of Oulu and Oulu University Hospital, 90220 Oulu, Finland; 7 Department of Biostatistics, University of Liverpool, Liverpool, L69 3GL UK; 8 Big Data Institute, Li Ka Shing Center for Health Information and Discovery, Oxford University, Oxford, OX3 7LF UK; 9 Vanderbilt Genetics Institute, Vanderbilt Epidemiology Center, Institute for Medicine and Public Health, Department of Obstetrics and Gynecology, Vanderbilt University Medical Center, Nashville, TN 37203 USA; 10 Division of Epidemiology, Department of Medicine, Institute for Medicine and Public Health, Vanderbilt Genetics Institute, Vanderbilt University Medical Center, Nashville, TN 37203 USA; 11 Institute for Molecular Bioscience, University of Queensland, Brisbane, QLD 4072 Australia; 12 MRC Epidemiology Unit, University of Cambridge School of Clinical Medicine, Institute of Metabolic Science, Cambridge Biomedical Campus, Cambridge, CB2 0QQ UK; 13 Center for Life Course Health Research, Faculty of Medicine, University of Oulu, 90220 Oulu, Finland; 14 Unit of Primary Health Care, Oulu University Hospital, 90220 Oulu, Finland; 15 Department of Epidemiology and Biostatistics, MRC-PHE Centre for Environment and Health, School of Public Health, Imperial College London, London, W2 1PG UK; 16 Biocenter Oulu, University of Oulu, 90220 Oulu, Finland; 17 Department of Life Sciences, College of Health and Life Sciences, Brunel University London, Uxbridge, Middlesex UB8 3PH UK; 18 Department of Human Genetics, David Geffen School of Medicine, University of California at Los Angeles, Los Angeles, CA 90095 USA; 19 Division of Preventative Medicine, Brigham and Women’s Hospital, Harvard Medical School, Boston, MA USA; 20 Obstetrics and Gynecology Epidemiology Center, Brigham and Women’s Hospital and Harvard Medical School, Boston, MA 02115 USA; 21 Department of Epidemiology, Harvard T.H. Chan School of Public Health, Boston, MA 02115 USA; 22 Genetic Epidemiology, QIMR Berghofer Medical Research Institute, Brisbane, QLD 4006 Australia; 23 Psychiatric Genetics, QIMR Berghofer Medical Research Institute, Brisbane, QLD 4006 Australia; 24 Institute of Health and Biomedical Innovation and School of Biomedical Science, Queensland University of Technology, Brisbane, QLD 4059 Australia; 25 23andMe, Mountain View, CA 94041 USA; 26 Department of Obstetrics, Gynecology, and Reproductive Biology, College of Human Medicine, Michigan State University, Grand Rapids, MI 49503 USA; 27 Department of Pathology, Brigham and Women’s Hospital, Harvard Medical School, Boston, MA 02115 USA; 28 Broad Institute of MIT and Harvard, Cambridge, MA 02142 USA; 29 Manchester Centre for Audiology and Deafness, Manchester Academic Health Science Center, University of Manchester, Manchester, M13 9PL UK

**Keywords:** Genome-wide association studies, Reproductive biology, Epidemiology

Correction to: *Nature Communications* 10.1038/s41467-019-12536-4, published online 24 October 2019

The original version of this Article contained an error in Table 1, in which the odds ratios were incorrectly calculated. The correct version of Table 1 is:

**Table 1:** Overview of lead SNPs with significant associations at 29 independent loci in UL GWAS meta-analysis

Locus	Lead SNP	RA	OA	RAF_EUR_	*P*_Meta_	OR (95% CI)	Gene(s) of interest^a^
1p36.12^b,c^	rs7412010	C	G	0.15	2.4 × 10^−29^	1.13 (1.11–1.16)	*WNT4, CDC42*
2p23.2	rs55819434	A	G	0.91	5.6 × 10^−09^	0.92 (0.90–0.95)	*BABAM2*
2p25.1^b,c^	rs35417544	T	C	0.69	2.3 × 10^−19^	1.09 (1.07–1.10)	*GREB1*
3q26.2^c^	rs35446936	A	G	0.24	1.0 × 10^−08^	0.95 (0.93–0.96)	*TERC*
4q12^c^	rs62323682	T	C	0.94	4.9 × 10^−18^	0.87 (0.84–0.90)	*LNX1, PDGFRA*
4q13.3^c^	rs12640488	A	G	0.52	4.0 × 10^−14^	0.94 (0.92–0.96)	*SULT1B1*
4q22.3	rs4699299	T	C	0.69	4.7 × 10^−08^	0.95 (0.94–0.97)	*PDLIM5*
5p15.33^c^	rs72709458	T	C	0.23	4.7 × 10^−21^	1.10 (1.08–1.13)	*TERT*
5q35.2^c^	rs2456181	C	G	0.49	1.1 × 10^−11^	0.94 (0.93–0.96)	*ZNF346, UIMC1*
6p21.31	rs116251328	A	T	0.02	3.0 × 10^−08^	1.15 (1.09–1.21)	*GRM4, HMGA1*
6q25.2^b,c^	rs58415480	C	G	0.84	1.9 × 10^−54^	0.84 (0.82–0.86)	*SYNE1, ESR1*
7q31.2	rs2270206	A	C	0.16	4.6 × 10^−08^	1.06 (1.04–1.09)	*WNT2*
9p24.3^c^	rs10976689	A	G	0.60	2.4 × 10^−13^	0.94 (0.93–0.96)	*ANKRD15*
10q24.3^c^	rs9419958	T	C	0.13	1.1 × 10^−16^	1.10 (1.08–1.13)	*OBFC1, SLK*
10p11.22	rs10508765	A	G	0.80	1.5 × 10^−10^	1.07 (1.05–1.09)	*ZEB1, ARHGAP12*
11p15.5^c^	rs547025	T	C	0.92	1.5 × 10^−14^	1.13 (1.09–1.16)	*RIC8A, BET1L*
11p14.1^b^	rs11031006	A	G	0.14	5.7 × 10^−15^	0.91 (0.89–0.93)	*FSHB*
11p13^c^	rs61889186	C	G	0.86	1.4 × 10^−25^	0.89 (0.87–0.91)	*WT1*
11p13^c^	rs2785202	C	G	0.55	6.9 × 10^−14^	1.06 (1.05–1.08)	*PDHX, CD44*
11q22.3^c^	rs149934734	T	C	0.03	1.1 × 10^−27^	1.33 (1.26–1.40)	*C11orf65, KDELC2*
12q13.11^c^	rs2131371	A	C	0.28	1.6 × 10^−18^	0.93 (0.91–0.94)	*SLC38A2*
12q15	rs11178393	T	C	0.89	3.3 × 10^−08^	1.08 (1.05–1.10)	*PTPRR*
12q24.31	rs28583837	A	G	0.22	2.3 × 10^−08^	0.94 (0.92–0.96)	*PITPNM2*
13q14.11^c^	rs117245733	A	G	0.02	5.7 × 10^−14^	1.31 (1.21–1.39)	*FOXO1*
17p13.1^c^	rs78378222	T	G	0.99	7.1 × 10^−31^	0.65 (0.60–0.70)	*SHBG, TP53*
20p12.3^c^	rs16991615	A	G	0.07	8.8 × 10^−10^	1.11 (1.07–1.14)	*MCM8, TRMT6*
22q13.1^c^	rs4821939	A	T	0.20	7.8 × 10^−16^	1.08 (1.06–1.10)	*TNRC6B*
Xp26.2^c^	rs12392108	A	T	0.31	5.9 × 10^−46^	1.13 (1.11–1.15)	*RAP2C*
Xq13.1^c^	rs4360450	A	G	0.37	2.1 × 10^−18^	1.08 (1.06–1.10)	*MED12*

*SNP* single-nucleotide polymorphism, *RA* risk allele, *OA* other allele, *RAF*_*EUR*_ average risk allele frequency in European samples, *OR* odds ratio

^a^≤300 kb distant from association signal

^b^Loci previously associated with endometriosis

^c^Loci previously associated with UL

which replaces the previous incorrect version:

**Table 1:** Overview of lead SNPs with significant associations at 29 independent loci in UL GWAS meta-analysis

Locus	Lead SNP	RA	OA	RAF_EUR_	*P*_Meta_	OR (95% CI)	Gene(s) of interest^a^
1p36.12^b,c^	rs7412010	C	G	0.15	2.4 × 10^−29^	1.13 (1.11–1.16)	*WNT4, CDC42*
2p23.2	rs55819434	A	G	0.91	5.6 × 10^−09^	1.09 (1.06–1.12)	*BABAM2*
2p25.1^b,c^	rs35417544	T	C	0.69	2.3 × 10^−19^	1.09 (1.07–1.10)	*GREB1*
3q26.2^c^	rs35446936	A	G	0.24	1.0 × 10^−08^	1.06 (1.04–1.08)	*TERC*
4q12^c^	rs62323682	T	C	0.94	4.9 × 10^−18^	1.15 (1.12–1.19)	*LNX1, PDGFRA*
4q13.3^c^	rs12640488	A	G	0.52	4.0 × 10^−14^	1.06 (1.05–1.08)	*SULT1B1*
4q22.3	rs4699299	T	C	0.69	4.7 × 10^−08^	1.05 (1.03–1.07)	*PDLIM5*
5p15.33^c^	rs72709458	T	C	0.23	4.7 × 10^−21^	1.10 (1.08–1.13)	*TERT*
5q35.2^c^	rs2456181	C	G	0.49	1.1 × 10^−11^	1.06 (1.04–1.08)	*ZNF346, UIMC1*
6p21.31	rs116251328	A	T	0.02	3.0 × 10^−08^	1.15 (1.09–1.21)	*GRM4, HMGA1*
6q25.2^b,c^	rs58415480	C	G	0.84	1.9 × 10^−54^	1.19 (1.17–1.22)	*SYNE1, ESR1*
7q31.2	rs2270206	A	C	0.16	4.6 × 10^−08^	1.06 (1.04–1.09)	*WNT2*
9p24.3^c^	rs10976689	A	G	0.60	2.4 × 10^−13^	1.06 (1.05–1.08)	*ANKRD15*
10q24.3^c^	rs9419958	T	C	0.13	1.1 × 10^−16^	1.10 (1.08–1.13)	*OBFC1, SLK*
10p11.22	rs10508765	A	G	0.80	1.5 × 10^−10^	1.07 (1.05–1.09)	*ZEB1, ARHGAP12*
11p15.5^c^	rs547025	T	C	0.92	1.5 × 10^−14^	1.13 (1.09–1.16)	*RIC8A, BET1L*
11p14.1^b^	rs11031006	A	G	0.14	5.7 × 10^−15^	1.10 (1.07–1.12)	*FSHB*
11p13^c^	rs61889186	C	G	0.86	1.4 × 10^−25^	1.12 (1.10–1.15)	*WT1*
11p13^c^	rs2785202	C	G	0.55	6.9 × 10^−14^	1.06 (1.05–1.08)	*PDHX, CD44*
11q22.3^c^	rs149934734	T	C	0.03	1.1 × 10^−27^	1.33 (1.26–1.40)	*C11orf65, KDELC2*
12q13.11^c^	rs2131371	A	C	0.28	1.6 × 10^−18^	1.08 (1.06–1.10)	*SLC38A2*
12q15	rs11178393	T	C	0.89	3.3 × 10^−08^	1.08 (1.05–1.10)	*PTPRR*
12q24.31	rs28583837	A	G	0.22	2.3 × 10^−08^	1.06 (1.04–1.08)	*PITPNM2*
13q14.11^c^	rs117245733	A	G	0.02	5.7 × 10^−14^	1.31 (1.21–1.39)	*FOXO1*
17p13.1^c^	rs78378222	T	G	0.99	7.1 × 10^−31^	1.54 (1.43–1.66)	*SHBG, TP53*
20p12.3^c^	rs16991615	A	G	0.07	8.8 × 10^−10^	1.11 (1.07–1.14)	*MCM8, TRMT6*
22q13.1^c^	rs4821939	A	T	0.20	7.8 × 10^−16^	1.08 (1.06–1.10)	*TNRC6B*
Xp26.2^c^	rs12392108	A	T	0.31	5.9 × 10^−46^	1.13 (1.11–1.15)	*RAP2C*
Xq13.1^c^	rs4360450	A	G	0.37	2.1 × 10^−18^	1.08 (1.06–1.10)	*MED12*

*SNP* single-nucleotide polymorphism, *RA* risk allele, *OA* other allele, *RAF*_*EUR*_ average risk allele frequency in European samples, *OR* odds ratio

^a^≤300 kb distant from association signal

^b^Loci previously associated with endometriosis

^c^Loci previously associated with UL

This has been corrected in both the PDF and HTML versions of the Article.

