# Supplementary material for: Genome-wide association and epidemiological analyses reveal common genetic origins between uterine leiomyomata and endometriosis
Source: Nat Commun. 2019 Oct 24;10:4857. doi: 10.1038/s41467-019-12536-4 (PMC6813337; doi:10.1038/s41467-019-12536-4)
Supplement: Supplementary file 3 — Reporting Summary [file 41467_2019_12536_MOESM3_ESM.pdf]

Description of Additional Supplementary Files

Supplementary Data 1. LD Hub analysis. LD Hub analysis was performed for various cardiometabolic, anthropometric, glycemic, blood lipids and reproductive diseases/traits.

Supplementary Data 2. DEPICT analyses. Independent loci identified from 8,971 SNPs with suggestive ($P < 1 \times 10^{-5}$) or significant associations ($P < 5 \times 10^{-8}$) for DEPICT analyses.

Supplementary Data 3. Gene-set enrichment analysis. Significant (FDR < 0.05) gene-sets determined from DEPICT-based gene-set enrichment analysis of 104 independent lead SNPs identified from 8,971 SNPs with suggestive ($P < 1 \times 10^{-5}$) or significant associations ($P < 5 \times 10^{-8}$).

Supplementary Data 4. Summary-data based Mendelian Randomization (SMR) using eQTL data from peripheral blood. This table includes all significant hits according to the SMR test suggesting a causal/pleiotropic association between the variant, disease risk, and gene expression. All hits that also passed the HEIDI test (test for heterogeneity) are highlighted in yellow.

Supplementary Data 5. Summary-data based Mendelian Randomization (SMR) using mQTL data from peripheral blood. This table includes all significant hits according to the SMR test suggesting a causal/pleiotropic association between the variant, disease risk, and gene methylation. All hits that passed also the HEIDI test (test for heterogeneity) are highlighted in yellow.

Supplementary Data 6. Top 10,000 genetic variants from the UL GWAS meta-analysis. Summary statistics for 10,000 genetic variants from the METAL meta-analysis of WGHS, NFBC, QIMR, UKBB and 23andMe. CHR = Chromosome; BP = base pair position; A1 = reference allele; A2 = alternate allele; BETA = effect size w/ respect to A1; BETA_SE = standard error of beta; P = significance of association; Freq1 = frequency of A1 allele; FreqSE = standard error of Freq1; HetISq = heterogeneity I-squared parameter; HetChiSq = heterogeneity chi-squared statistic; HetDf = heterogeneity degrees of freedom; HetP = significance of heterogeneity.
