# Supplementary material for: Genome-wide association and epidemiological analyses reveal common genetic origins between uterine leiomyomata and endometriosis
Source: Nat Commun. 2019 Oct 24;10:4857. doi: 10.1038/s41467-019-12536-4 (PMC6813337; doi:10.1038/s41467-019-12536-4)
Supplement: Supplementary file 10 — Supplementary Data 6 [file 41467_2019_12536_MOESM10_ESM.pdf]

Supplementary information

Genome-wide association and epidemiological analyses reveal common genetic origins between uterine leiomyomata and endometriosis

Gallagher et al.

Supplementary Methods

GWAS – FibroGENE Cohort Descriptions

Women's Genome Health Study (WGHS)

WGHS is a nested cohort within the Women's Health Study (WHS)[1], an ongoing prospective cohort that was originally launched in 1992 as a randomized controlled trial of female North American health-care professionals focusing on cardiovascular and cancer outcomes, who provided a blood sample at baseline and consented for blood-based analyses. All participants were at least 45 years of age and free of cardiovascular disease, cancer, or other major chronic illnesses at the time of consent. Health- and lifestyle-related information were collected via questionnaires at enrollment and follow-up time points. WHS participants were asked whether they had ever been diagnosed with UL and their age at diagnosis. Cases were defined as women who self-reported 'yes' to having a history of UL, while controls were classified as women who self-reported 'no'. Women who reported an age of UL diagnosis < 20 or > 70 years of age were excluded from the analysis. Participants in WGHS were recruited under an IRB-approved protocol by the Partners HealthCare System Human Research Committee. For this study, a total of 12,840 women of white European ancestry were included: 3,375 UL cases and 9,465 controls.

Northern Finland Birth Cohort (NFBC)

NFBC includes two longitudinal and prospective birth cohorts of white European women and offspring collected at 20-year intervals from the same provinces of Oulu and Lapland in Finland: NFBC1966 and NFBC1986. In this study, we utilized data from NFBC1966. Cases (*n*=363) with

a history of UL were identified through national outpatient and inpatient hospital discharge registers and self-reported diagnosis through postal questionnaire at age 46. The hospital discharge registers include WHO ICD codes for identification of disease diagnoses and dates for each hospital visit. Controls (*n*=5,000) were drawn from the rest of the cohort population. Informed consent was obtained from all participants using protocols approved by the Ethical Committee of the Northern Ostrobothnia Hospital District.

QIMR Berghofer Medical Research Institute (QIMR)

In the QIMR cohort women were originally recruited into a study examining predisposition to endometriosis[2] and a twin study of gynecological health[3]. The cohort includes affected sister pair families (aged 15-87 years [affected women] at the end of sample collection) and twin pairs (aged 29-91 years at the return of questionnaire) of white European women. For both studies, women completed questionnaires on various aspects of their reproductive health. Participants who answered “yes” to the “uterine fibroids” option of the question “Have you ever had any of the following conditions?” were selected as cases (*n*=1,484). Of the 1484 cases, 585 came from the endometriosis sample, 579 had a surgically-confirmed diagnosis of endometriosis, the remaining six had a family member (daughter, cousin, or 2nd cousin) diagnosed with endometriosis. Controls (*n*=3,701) were drawn from twin pairs in the gynecological health study in which both sisters answered “no” to a question about medical history of uterine fibroids (one sample per twin pair). Validation of self-reported hysterectomy has previously been examined in the twin pairs; the diagnosis was confirmed in 97.6% of those who reported hysterectomy and for whom medical response from a physician was available[3]. Informed consent was obtained from all participants.

Approval for the studies was granted by the Human Research Ethics Committee at the QIMR Berghofer Medical Research Institute and the Australian Twin Registry.

UK Biobank (UKBB)

The UKBB is a large national and international health resource following the health and well-being of 500,000 male and female volunteer participants, enrolled at ages from 40 to 69[4]. The UKBB study began in 2006 with the aim to follow the participants for at least 30 years thereafter. Information has been collected from participants during recruitment using questionnaires on socioeconomic status, lifestyle, family history and medical history. Participants have also been followed up for cause-specific morbidity and mortality through linkage to disease registries, death registries, and hospital admission records. For this study, altogether 220,936 women of European ancestry were considered. Based on both hospital-linked medical records and self-report (interview with research nurse), women with a history of UL were selected as cases (*n*=15,184), while controls (*n*=205,752) had no previous history of UL. When limited by heavy menstrual bleeding (HMB), a total of 3,409 women with both UL and HMB were selected as cases, and 199,171 women as controls without a history of UL or HMB. For HMB, only cases with hospital-linked medical records were considered (*n*=9,813). Informed consent was obtained from all participants. The UKBB project is approved by the North West Multi-centre Research Ethics Committee.

23andMe Cohort

Participants were drawn from the customer base of 23andMe (Mountain View, CA, USA). For this study, the 23andMe cohort included 58,655 unrelated European women. Data on participants'

history of UL were collected via self-report in online surveys. Medical history of UL was determined with the research question, "Have you ever been diagnosed with uterine fibroids?", which had three response options: yes, no, and not sure. Females who answered "yes" were selected as cases, those who answered "no" as controls, and those who answered "not sure" were excluded from the study, resulting in 15,068 cases and 43,587 controls. All 23andMe research participants provided informed consent and answered surveys online according to a human subject protocol approved by Ethical and Independent Review Services, an external institutional review board.

Comorbidity analysis – Cohort descriptions and statistical analyses

Nurse's Health Study II (NHSII)

NHSII is an ongoing prospective cohort established in 1989 when 116,429 female registered nurses, aged 25 to 42 years, completed a baseline questionnaire on demographic and lifestyle factors, anthropometric variables, and disease histories. Starting in 1993, participants were asked if they had "ever had physician-diagnosed uterine fibroid(s)," and, if so, the date of diagnosis and whether it had been confirmed by pelvic exam or ultrasound/hysterectomy. Thus, the NHSII participants were queried about endometriosis diagnosis prospectively during reproductive years, in contrast to recalled diagnosis from often many years prior for all of WHS and the majority of UKBB participants. The validity of self-reported UL has previously been examined, with the diagnosis confirmed in 93% of women who reported ultrasound or hysterectomy confirmation[5]. Definition of UL diagnosis was restricted to women who reported ultrasound or hysterectomy confirmation of their diagnosis. Participants were also asked if they had "ever had physician-

diagnosed endometriosis," and, if so, the date of diagnosis and whether it had been confirmed by laparoscopy. The validity of self-reported endometriosis has previously been examined, with the diagnosis confirmed in only 54% of those without report of surgical confirmation but in 96% of these medical professional women who reported laparoscopic confirmation[6]. Therefore, cases of endometriosis were restricted to women who reported laparoscopic confirmation of their diagnosis.

Time-dependent survival analysis methods were applied to the NHSII prospective cohort data. Participants contributed follow-up time from the return of the 1989 questionnaire until report of UL, diagnosis of any cancer with the exception of non-melanoma skin cancer, hysterectomy, menopause, death, or loss to follow-up, whichever occurred first. No other censoring or exclusion criteria were applied. Cox proportional hazards regression models with age and questionnaire period as the time scale and time-varying covariates were used to estimate hazard rate ratios (HR) and 95% confidence intervals (CI) of ultrasound/hysterectomy confirmed UL in participants with laparoscopically confirmed endometriosis compared to those without. Both prevalent (diagnosed before the start of the cohort) and incident (diagnosed after study enrollment) cases of laparoscopically confirmed endometriosis were included. Time-varying covariates were updated in the analyses whenever new information was available from the biennial questionnaires. This study was approved by the Institutional Review Boards at Harvard T.H. Chan School of Public Health and Brigham and Women's Hospital (Partners Human Research Committee), Boston, MA.

Women's Health Study (WHS)

The full WHS cohort includes WGHS described above (WGHS, GWAS – FibroGENE Cohort Descriptions). Healthcare professionals in the WHS cohort encompass registered nurses, licensed practical nurses, licensed vocational nurses, physicians, veterinarians, pharmacists, dietitians, dentist, dental hygienists, speech/hearing/language professionals, physical therapists, and radiology technologists; thus, the overall medical training among the WHS participants in regard to gynecologic conditions was less certain compared to the NHSII participants. For the comorbidity analyses, all ancestries were included (i.e., there was not a restriction to European ancestry as was applied to the GWAS analyses). However, women who did not respond to at least one questionnaire that queried UL and endometriosis were excluded. Briefly, within the full WHS, UL cases included women who at enrollment in 1992 (when they were aged 45 years and older) or on subsequent questionnaires retrospectively self-reported having been diagnosed with UL between the ages of 20 and 70. Women who never reported UL at baseline or at any point during follow-up were classified as not having UL. WHS participants were asked on the 2009 questionnaire (when they were at least 60 years of age) if they ever had physician-diagnosed endometriosis, and if so, whether the diagnosis was confirmed by laparoscopy. As with NHSII, the case definition for the analysis was restricted to women who self-reported physician-diagnosed endometriosis with laparoscopic-confirmation of the diagnosis. The unexposed group was defined as participants who never self-reported having been diagnosed with endometriosis. Neither UL nor endometriosis reports from WHS participants have been validated.

No data on the age at or calendar time-period of endometriosis diagnosis were collected, and therefore for the WHS, cross-sectional logistic regression analyses were used to estimate odds

ratios (OR) and 95% CI for self-reported history of UL comparing those with a self-reported history of laparoscopically confirmed endometriosis to those without. The WHS cross-sectional analyses did not temporally order whether endometriosis was diagnosed before or after UL.

UK Biobank (UKBB)

The UKBB study is described in detail above (UK Biobank, GWAS – FibroGENE Cohort Descriptions). When the UKBB began in 2006, all participants were ≥ 40 years of age. UL and endometriosis cases were identified by retrospective self-reports (collected through questionnaires administered at recruitment by trained research nurses) and/or hospital diagnosis data (by linkage to hospital records with ICD-9/ICD-10 codes). The unexposed group was defined as participants who never self-reported having been diagnosed with endometriosis.

For the phenotypic comorbidity analyses all ancestries were included, with main groups based on self-report, including White European ancestry (95%), Asian (2%), Black, mixed, and other. Logistic regression models were used to estimate OR and 95% CI for self-reported history of UL comparing those with a documented history of endometriosis to those without. In this analysis, UL cases were identified by both self-reports and/or hospital diagnosis data, with hysterectomies being the main operation through which UL were diagnosed. However, for endometriosis, only self-reported diagnosis was used as the hospital diagnosis data were artificially inflated (likely due to the high number of hysterectomies resulting in diagnostic bias). As with the WHS analyses, the UKBB cross-sectional analyses did not consider temporality in UL and endometriosis diagnoses.

Covariates

Potential confounders included in multivariable models were defined as factors potentially associated with UL and/or endometriosis risk including: ancestry, age, body mass index (BMI), smoking status, age at menarche, oral contraceptive use, parity, age at first birth, menopausal status, use of anti-hypertensive medication/diastolic blood pressure, physical activity, and alcohol consumption. Risk factors included in models were defined to be consistent across cohorts whenever possible (see footnote in Table 2 for cohort-specific adjustment details), although the value of dynamic variables (*e.g.* age or BMI) was defined as time-varying and updated biennially in the NHSII cohort, while they were cross-sectionally defined at the time of data collection in WHS and UKBB.

Supplementary Note 1

WHS

WHS is supported by the National Heart, Lung, and Blood Institute (HL043851 and HL080467) and the National Cancer Institute (CA047988 and UM1CA182913) with funding for genotyping provided by Amgen.

NFBC

NFBC1966 received financial support related to this study from the Academy of Finland (project grants 104781, 120315, 129269, 1114194, 24300796, 85547, Center of Excellence in Complex Disease Genetics), University Hospital Oulu, Biocenter, University of Oulu, Finland (75617), NHLBI grant HL087679-02 through the STAMPEED program (MH083268), NIH/NIMH (MH63706), the EU FP5 EURO-BLCS, QLG1-CT-2000-01643, ENGAGE project and grant agreement HEALTH-F4-2007-201413, EU FP7 EurHEALTHAgeing 277849, the Medical Research Council (MRC), UK (G0500539, G0600705, G1002319, PrevMetSyn/SALVE) and ERDF European Regional Development Fund Grant no. 539/2010 A31592. The program is currently funded by the EU H2020-PHC-2014 DynaHEALTH action (grant agreements No. 633595), EU H2020-HCO-2004 iHEALTH Action, EU H2020-PHC-2014 ALEC Action (grant agreement No. 633212), EU H2020-SC1-2016-2017 LIFECYCLE Action, EU H2020-MSCA-ITN-2016 CAPICE Action, Academy of Finland EGEA-project (285547) and MRC Grant MR/M013138/1. DNA extractions, sample quality controls, biobank up-keeping, and aliquotting were performed in the National Public Health Institute, Biomedicum Helsinki, Finland and supported financially by the Academy of Finland and Biocentrum Helsinki. We thank the late

Professor Paula Rantakallio for the launch of NFBC1966. For further information, contact Professor Marjo-Riitta Jarvelin (m.jarvelin@imperial.ac.uk).

QIMR

Funding for the twin studies was provided by the Australian National Health and Medical Research Council (241944, 339462, 389927, 389875, 389891, 389892, 389938, 442915, 442981, 496739, 552485, 552498, 1084325), the Australian Research Council (A7960034, A79906588, A79801419, DP0770096, DP0212016, DP0343921), the FP-5 GenomEUtwin Project (QLG2-CT-2002-01254), and the U.S. National Institutes of Health (AA07535, AA10248, AA13320, AA13321, AA13326, AA14041, MH66206). The endometriosis study was supported by grants from the Australian National Health and Medical Research Council (241944, 339462, 389927,389875, 389891, 389892, 389938, 443036, 442915, 442981, 496610, 496739, 552485, 552498), the Cooperative Research Centre for Discovery of Genes for Common Human Diseases (CRC), Cerylid Biosciences (Melbourne), and donations from Neville and Shirley Hawkins.

UKBB

This research has been conducted using the UK Biobank Resource under application 9637 and was supported by the Medical Research Council [Unit Programme number MC_UU_12015/2].

NHSII

The Nurses' Health Study II is supported by the Public Health Service grant UM1 CA176726 from the National Cancer Institute and research grants HD4854, HD52473, HD57210, and HD081064 from the Eunice Kennedy Shriver National Institute of Child Health and Human Development.

Supplementary Figures and Tables

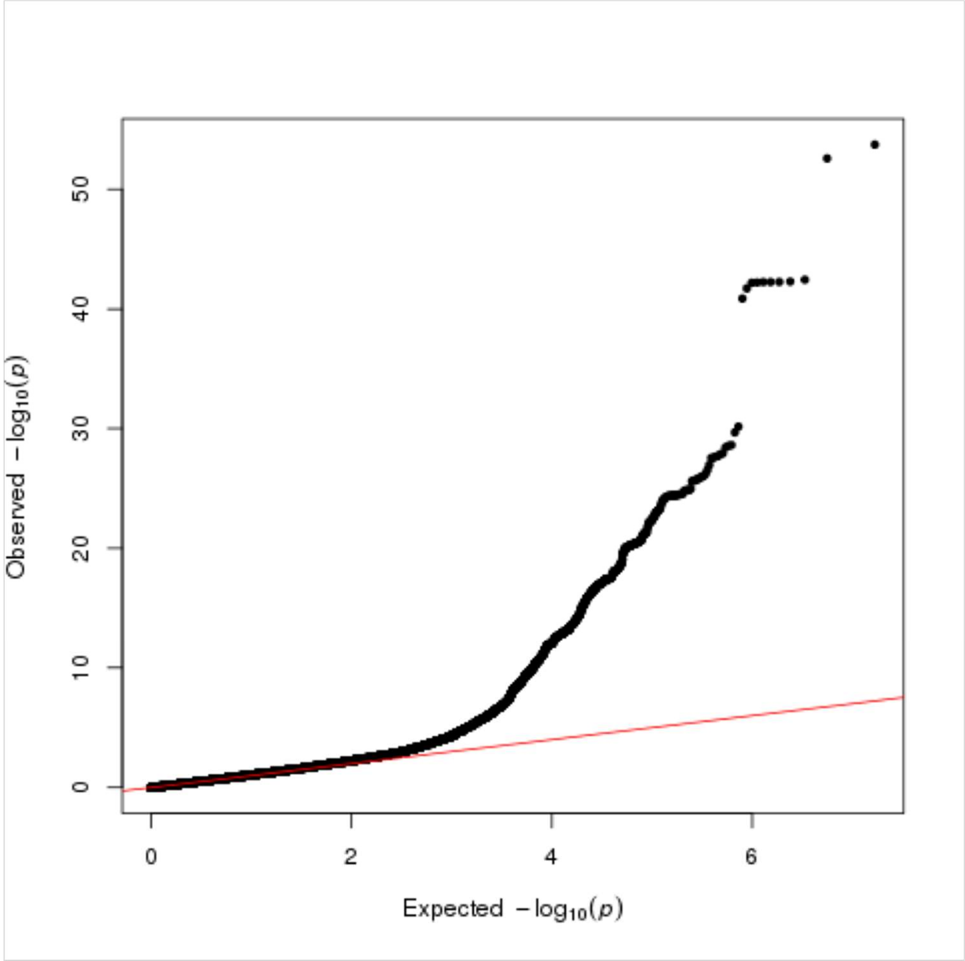

Supplementary Figure 1. Quantile-quantile plot. Quantile-quantile plot of *P*-values observed in meta-analysis of UL GWAS conducted in 302,979 women from the FibroGENE consortium. Genomic inflation factor (λ_{GC} = 1.042) indicates modest inflation in the χ^2 test statistic. The diagonal red line represents expected distribution of observed *P*-values under the null hypothesis of no association.

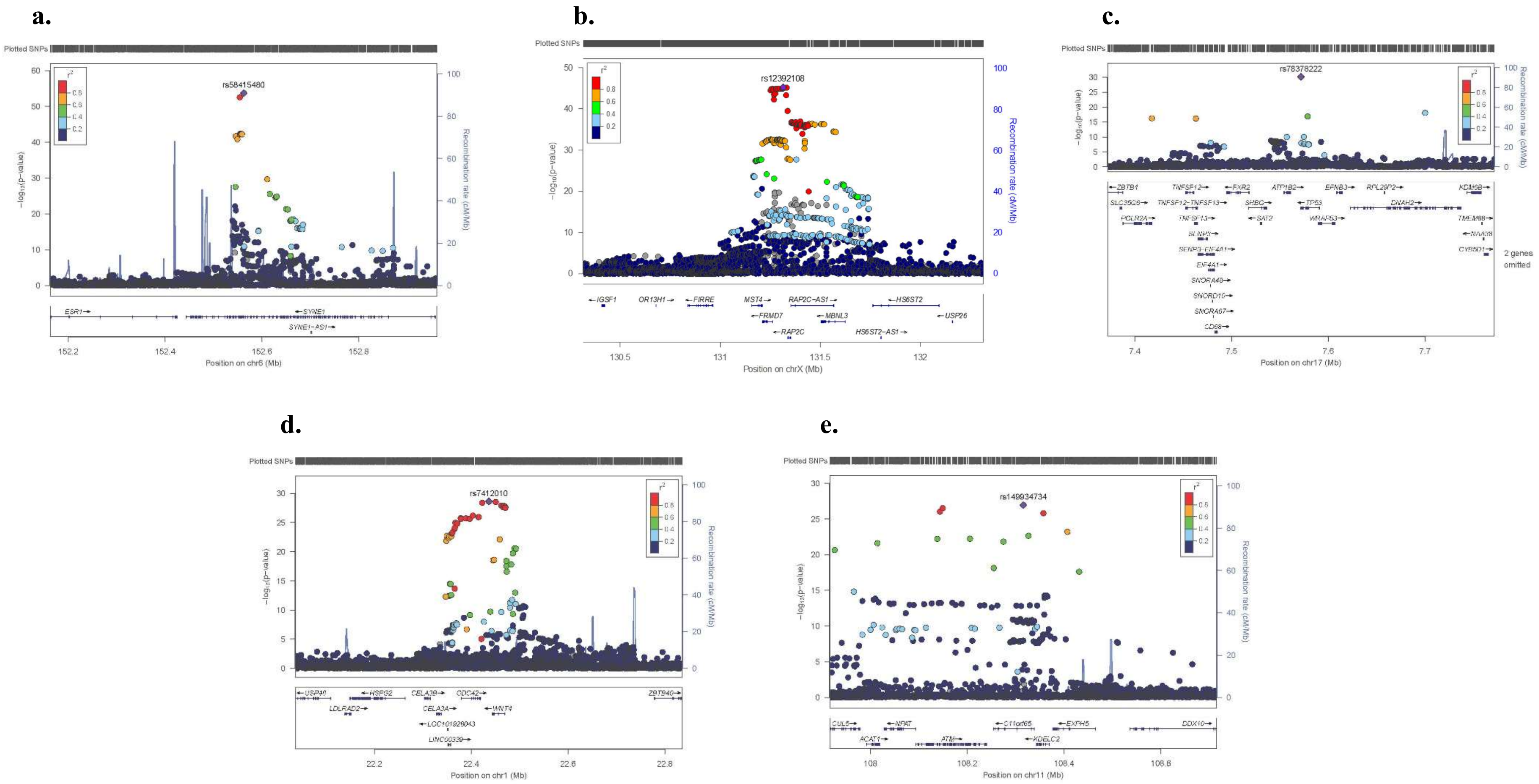

Supplementary Figure 2. Regional association plots for five top loci in the GWAS meta-analysis across all cohorts. Loci on chromosomes at: **a)** 6q25.2, **b)** Xp26.2, **c)** 17p13.1, **d)** 1p36.12, and **e)** 11q22.3. The labeled SNP represents the most significant SNP for each locus. SNP association *P*-value is shown on the y axis, while SNP position (with gene annotation) appears on the x axis. Each SNP is colored according to the strength of LD with the lead SNP. Plots were produced in LocusZoom.

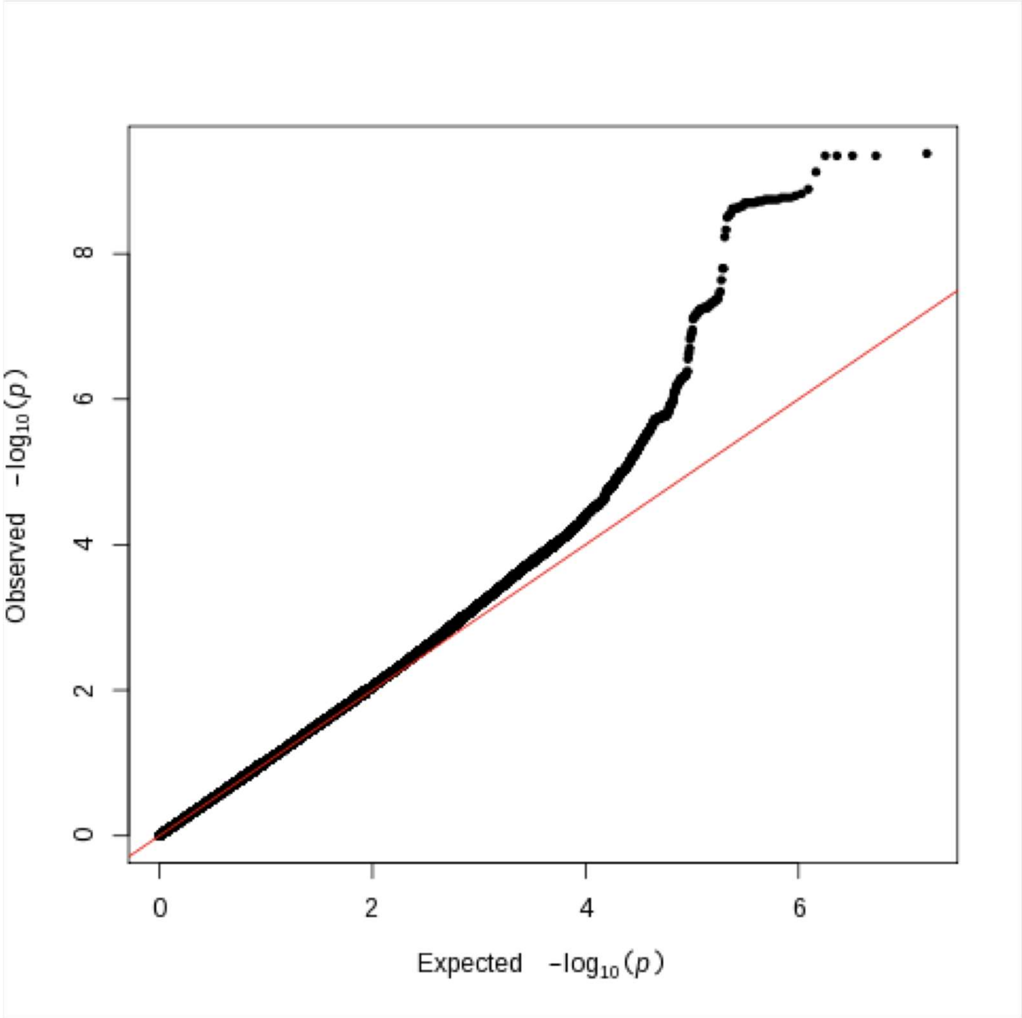

Supplementary Figure 3. Quantile-quantile plot. Quantile-quantile plot of *P*-values observed in UL limited by heavy menstrual bleeding GWAS conducted in 202,580 women from the UKBB. The diagonal red line represents expected distribution of observed *P*-values under the null hypothesis of no association.

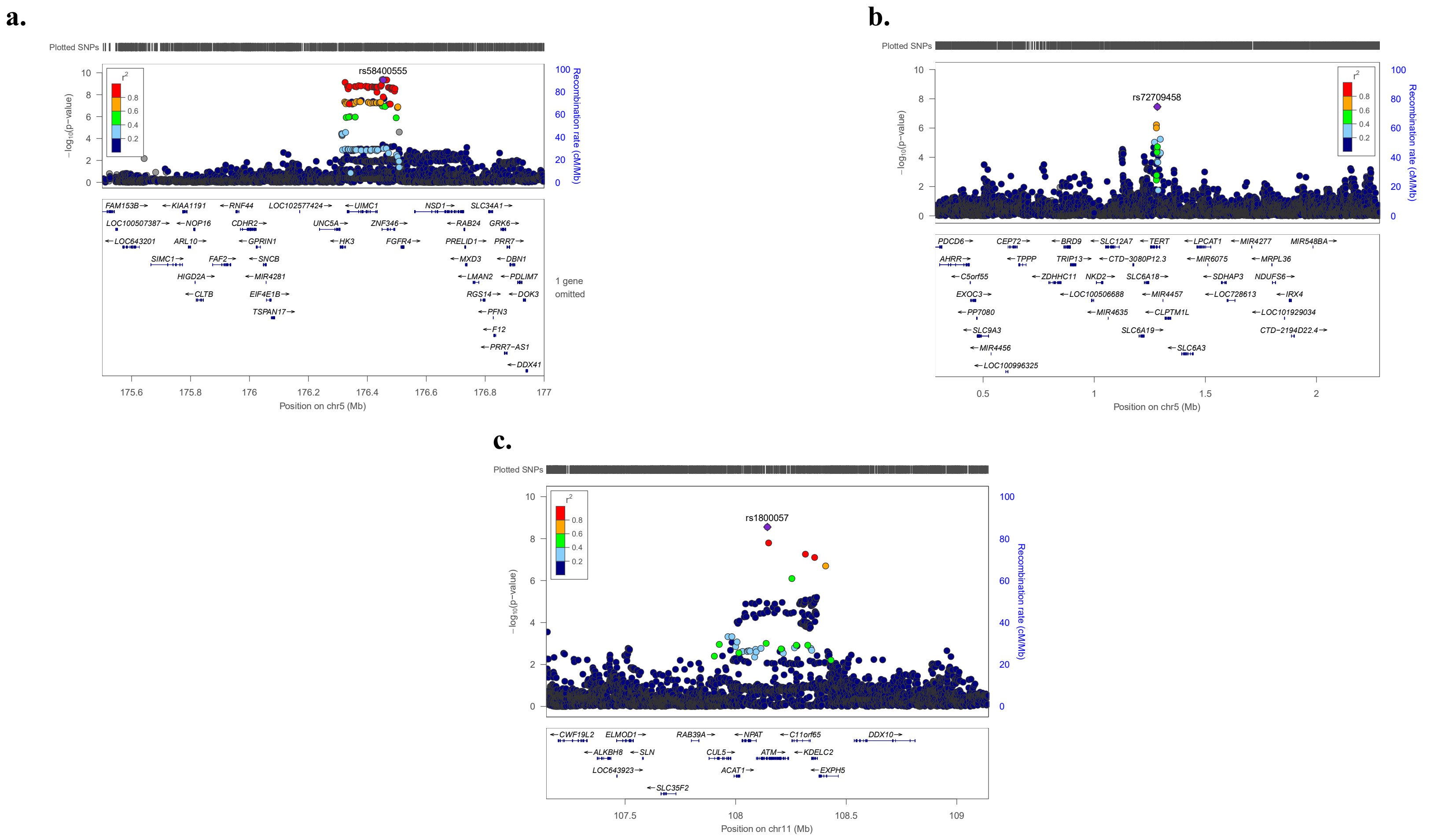

Supplementary Figure 4. Regional association plots for three genome-wide significant loci in the GWAS on UL limited by heavy menstrual bleeding. Loci on chromosomes at: **a)** 5q35.2, **b)** 5p15.33, and **c)** 11q22.3. The labeled SNP represents the most significant SNP for each locus except for 5q35.2, where the labeled SNP is the second most significant SNP. SNP association *P*-value is shown on the y axis, while SNP position (with gene annotation) appears on the x axis. Each SNP is colored according to the strength of LD with the lead SNP. Plots were produced in LocusZoom.

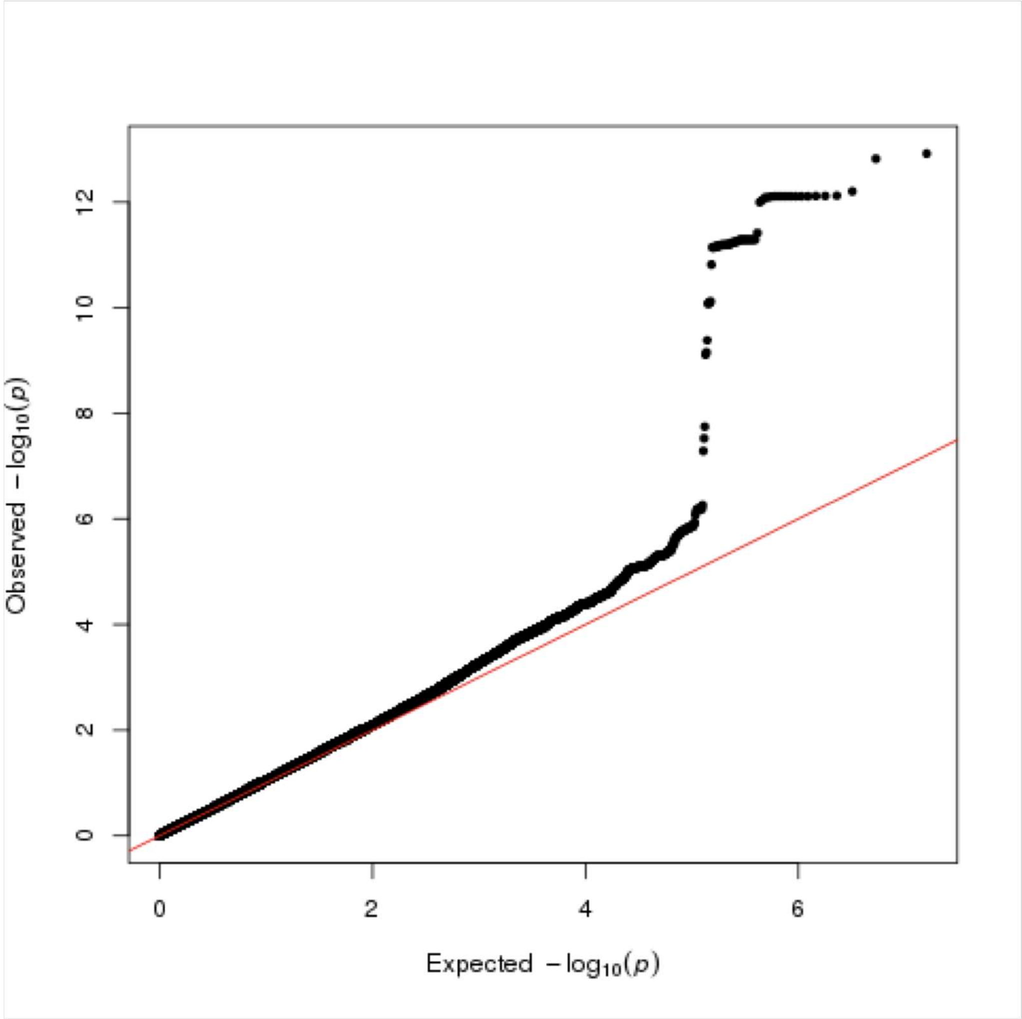

Supplementary Figure 5. Quantile-quantile plot. Quantile-quantile plot of *P*-values observed in heavy menstrual bleeding GWAS conducted in 220,759 women from the UKBB. The diagonal red line represents expected distribution of observed *P*-values under the null hypothesis of no association.

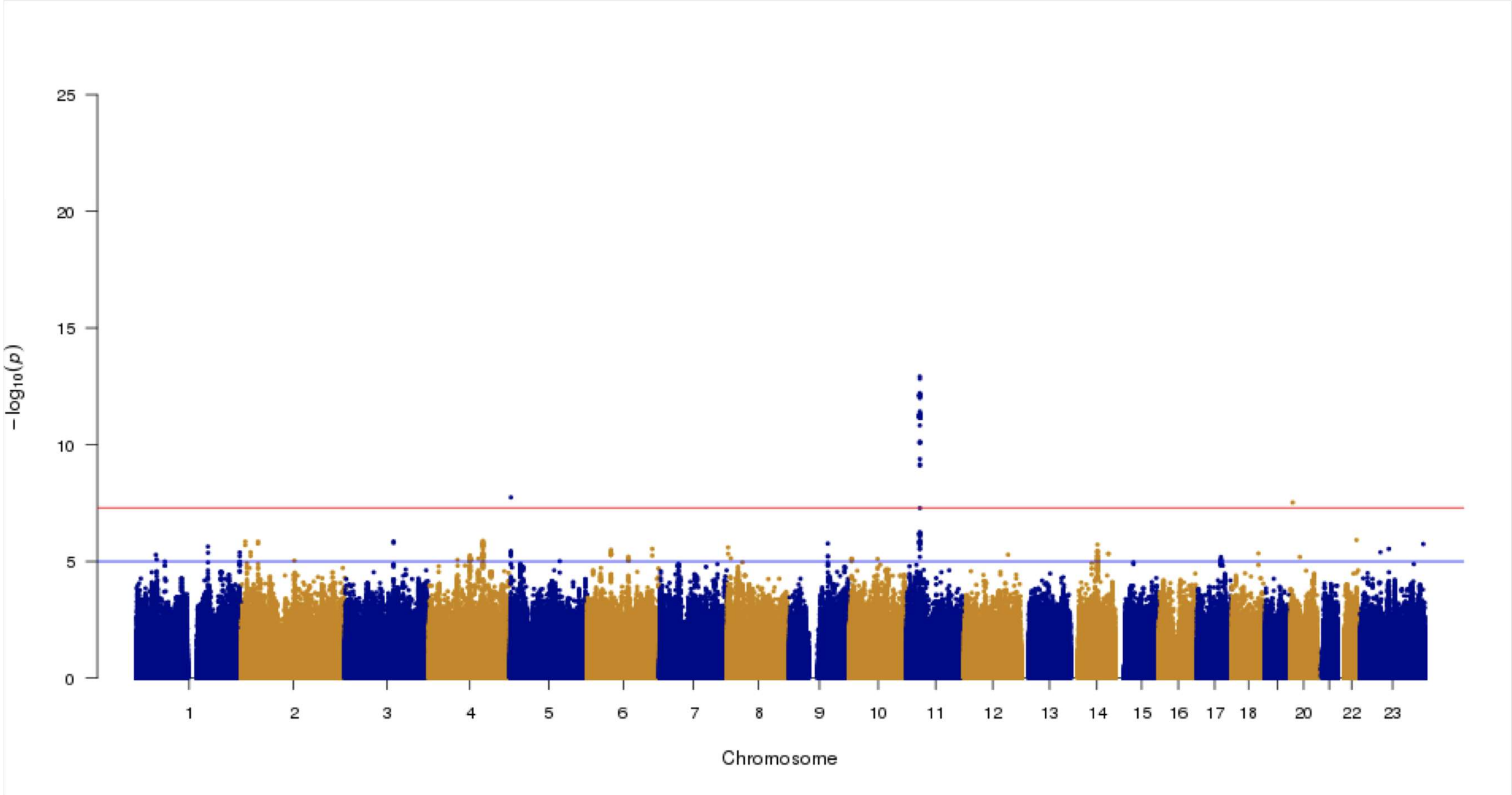

Supplementary Figure 6. Manhattan plot for heavy menstrual bleeding GWAS. GWAS across 220,759 women of white European reveals one genome-wide significant association at 11p14.1. Red and blue horizontal lines indicate genome-wide significant ($P < 5 \times 10^{-8}$) and suggestive ($P < 1 \times 10^{-5}$) thresholds, respectively.

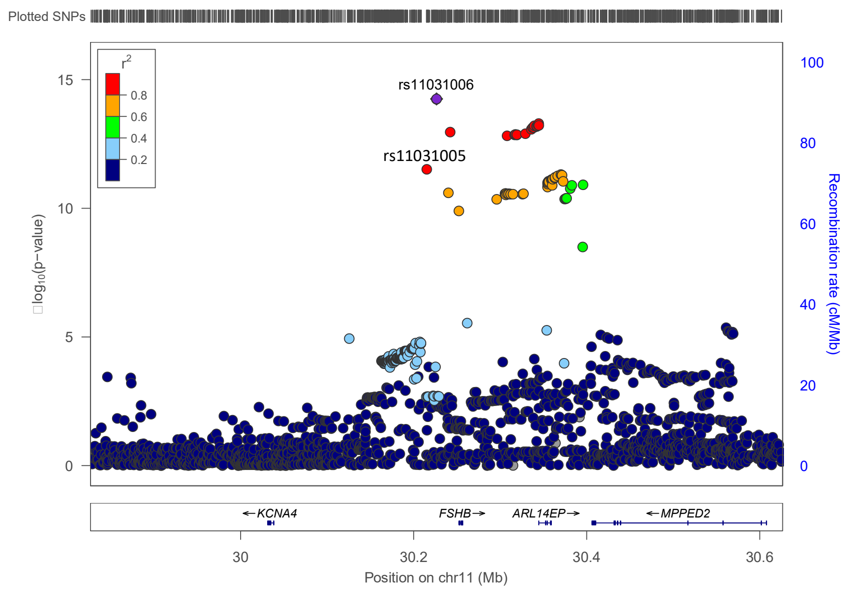

Chr location	UL lead SNP	RA	RAF_{EUR}	HMB lead SNP[a]	RA	RAF_{EUR}	r^2
11p14.1	rs11031006	A	0.14	rs11031005	C	0.14	1

RA, risk allele; RAF_{EUR}, average risk allele frequency in European samples; HMB, heavy menstrual bleeding

Supplementary Figure 7. Regional association plot and linkage disequilibrium at 11p14.1. Regional association plot for 11p14.1 in the GWAS meta-analysis on UL across all cohorts. The labeled SNP represents the most significant SNP for the locus. SNP association *P*-value is shown on the y axis, while SNP position (with gene annotation) appears on the x axis. Each SNP is colored according to the strength of LD with the lead SNP. Regional association plot was produced in LocusZoom. Also, linkage disequilibrium between the lead SNPs from UL GWAS meta-analysis and heavy menstrual bleeding GWAS at 11p14.1 in women of European ancestry is presented.

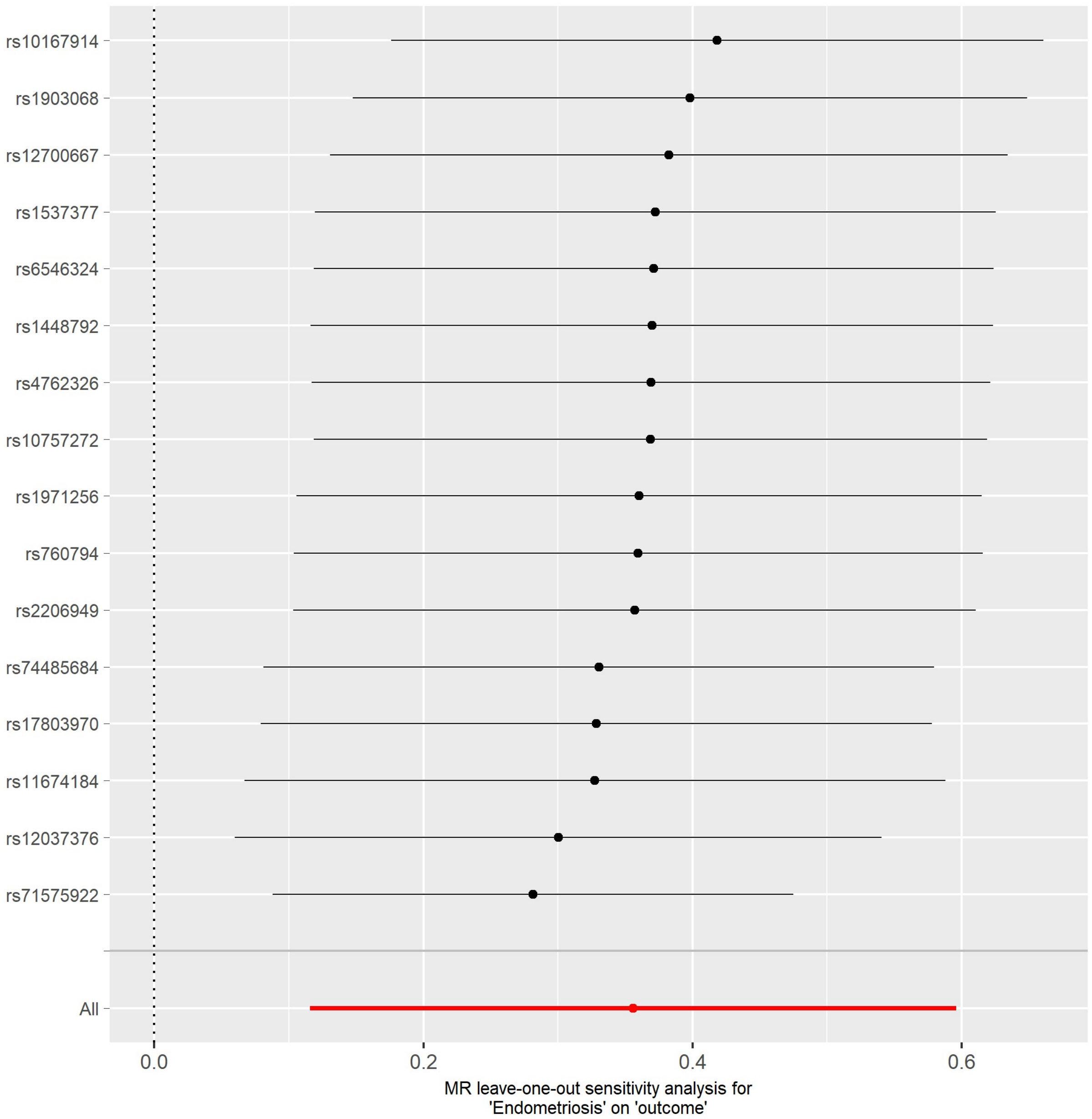

Supplementary Figure 8. Leave-one-out plot. Sixteen independent SNPs associated with endometriosis were available in the UL GWAS summary data to test for a causal effect of endometriosis on UL. Leave-one-out sensitivity analysis was performed to evaluate whether an individual variant was solely responsible for the significance of the observed relationship. Each black dot represents an IVW method for estimating the causal effect of endometriosis on UL. Error bars represent standard error (SE).

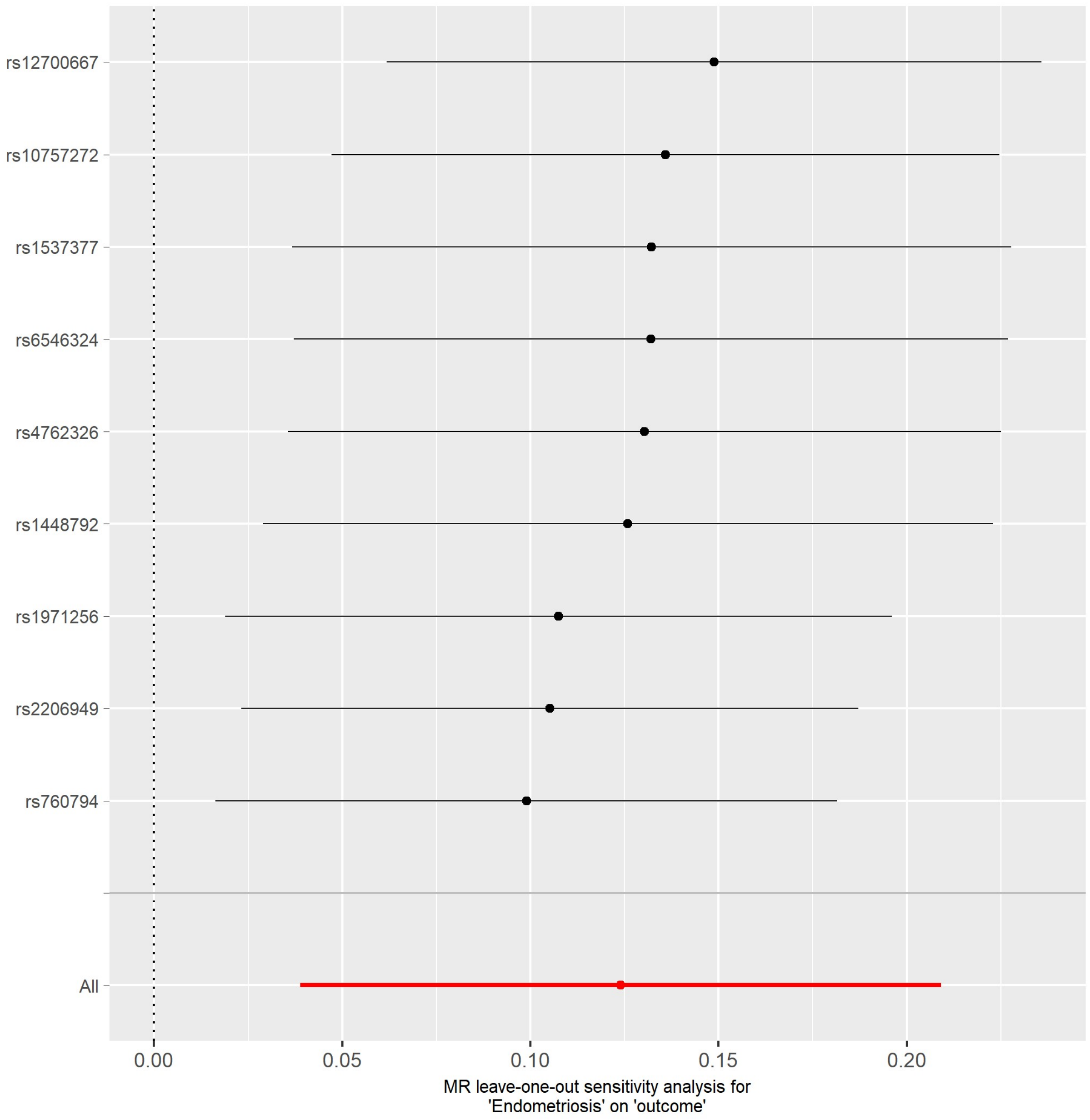

Supplementary Figure 9. Leave-one-out plot for the minimal set of variants. Nine independent SNPs associated with endometriosis were used to test for a causal effect of endometriosis on UL. Leave-one-out sensitivity analysis was performed to evaluate whether an individual variant was solely responsible for the significance of the observed relationship. Each black dot represents an IVW method for estimating the causal effect of endometriosis on UL. Error bars represent standard error (SE).

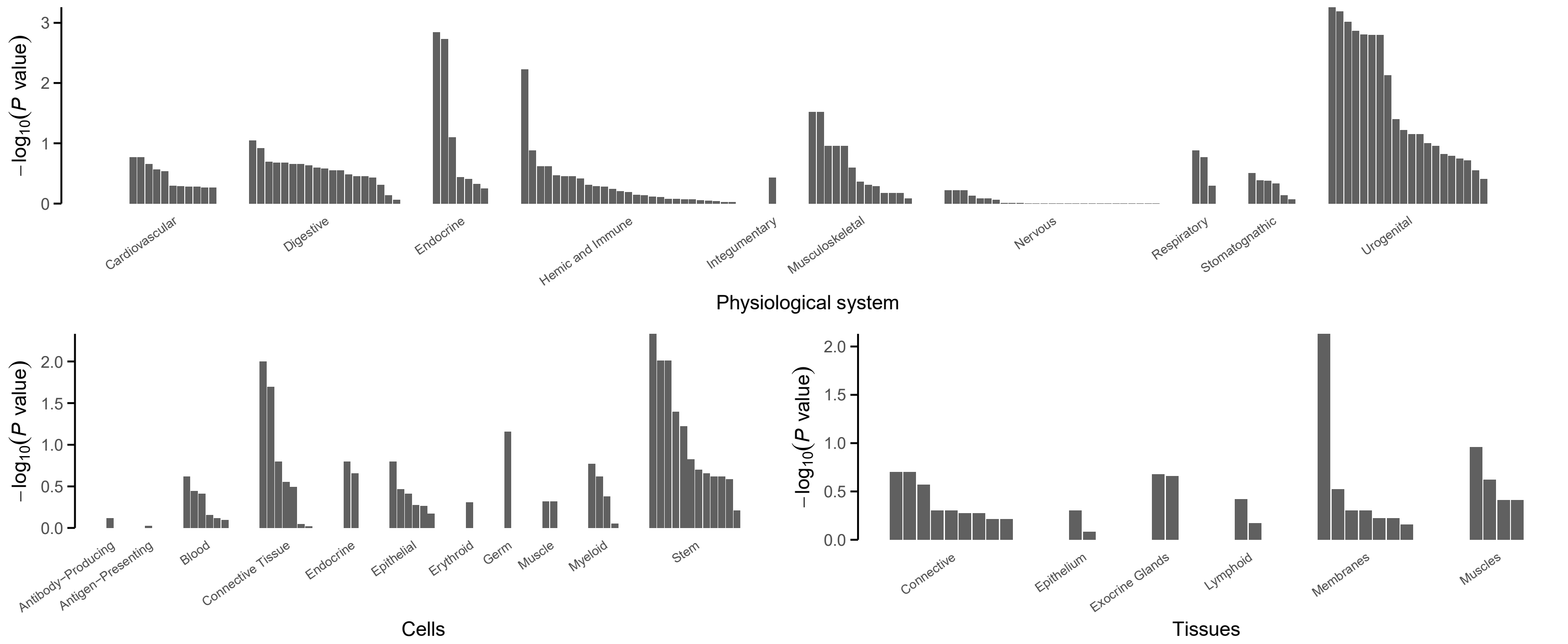

Supplementary Figure 10. Cell/tissue enrichment analysis. Results from DEPICT-based tissue enrichment analysis of 104 independent lead SNPs identified from 8,971 SNPs with suggestive ($P < 1 \times 10^{-5}$) or significant associations ($P < 5 \times 10^{-8}$). Of note, scales of the y axis differ between plots.

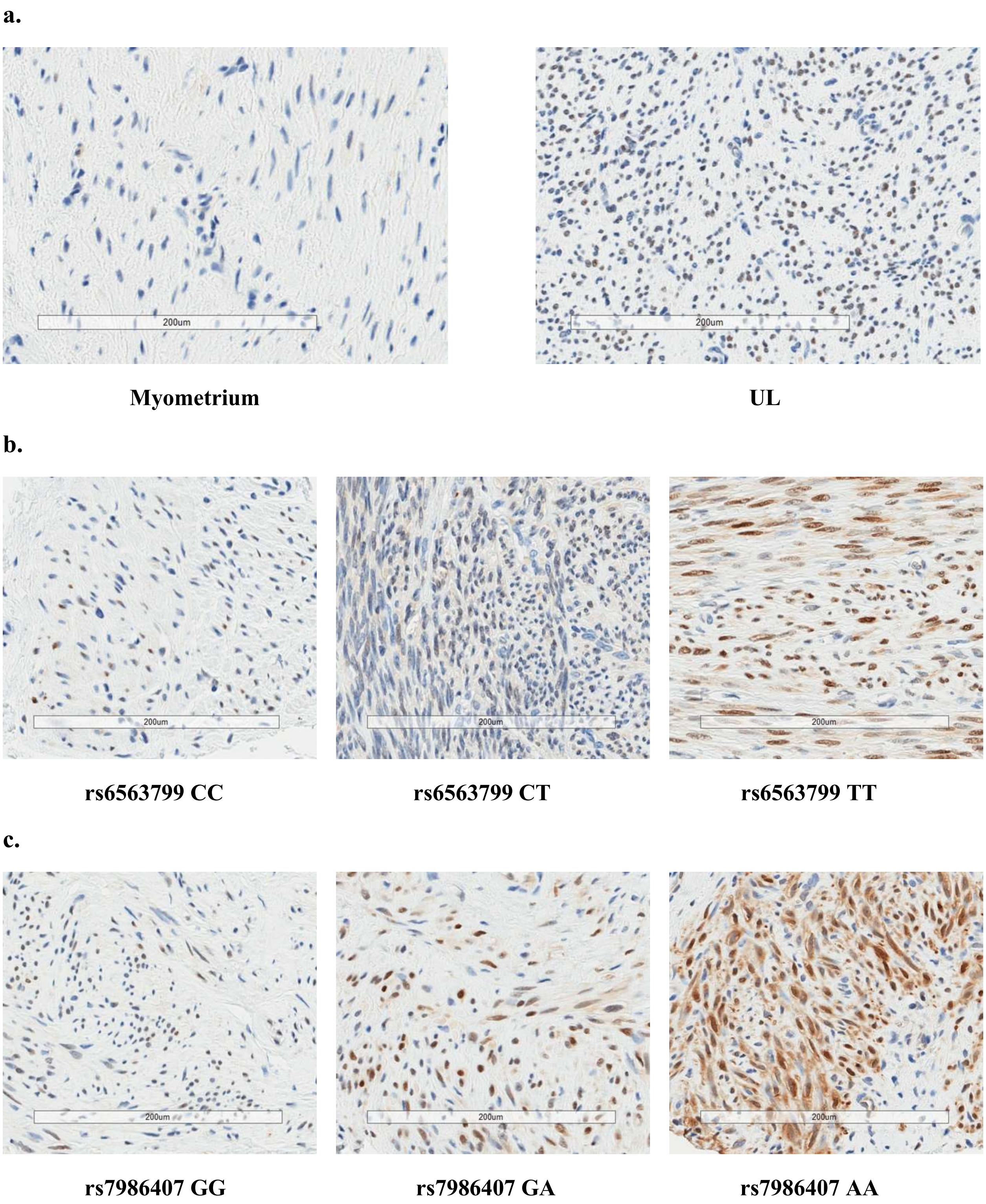

Supplementary Figure 11. Representative images of FOXO1 immunostainings. a) Nuclear expression of FOXO1 in a patient-matched UL and myometrial sample pair. **b)** Nuclear expression of FOXO1 in UL representing different genotypes of rs6563799. **c)** Nuclear expression of FOXO1 in UL representing different genotypes of rs7986407. The scale bar is 200µm.

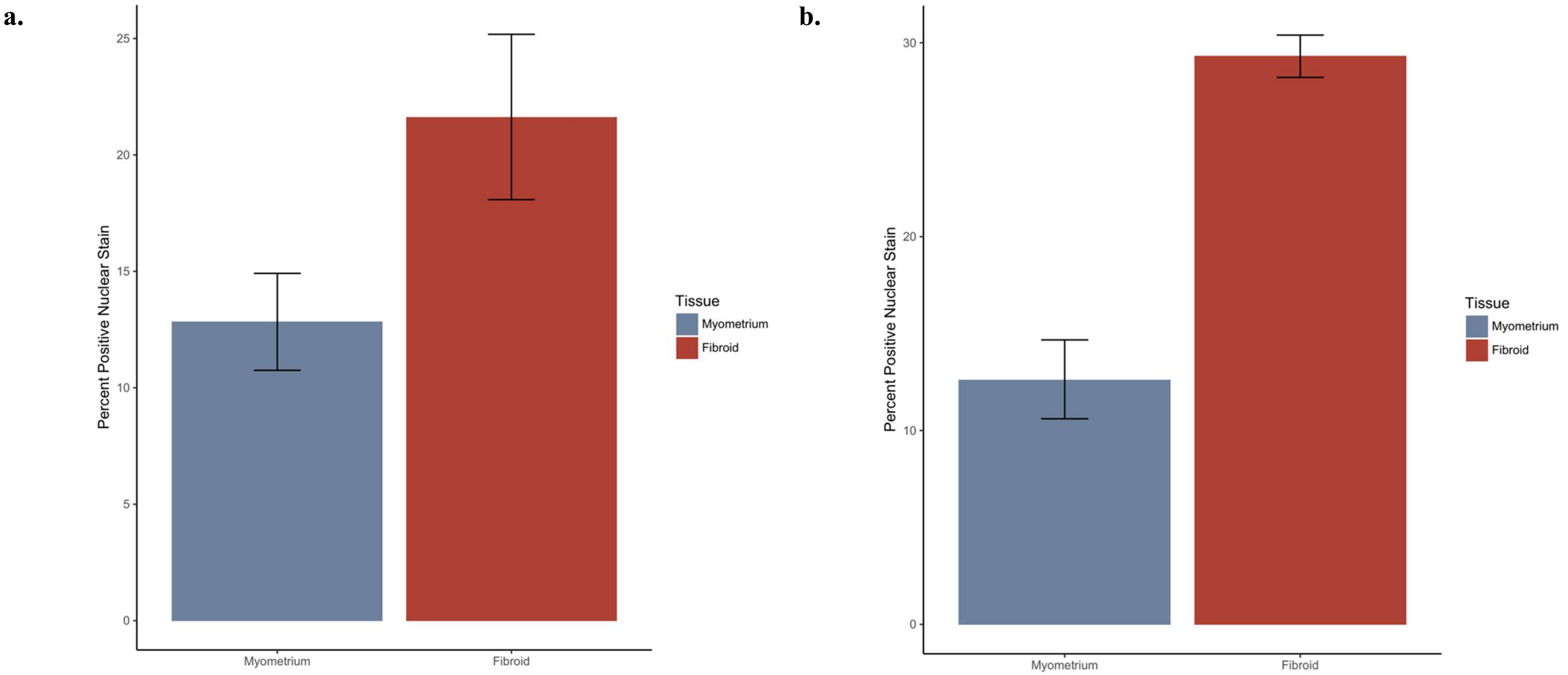

Supplementary Figure 12. FOXO1 immunostaining. **a)** Nuclear expression of FOXO1 is 1.69-fold greater in patient-matched UL and myometrial sample pairs. Percent of positive nuclei were quantified in 40 UL and 34 myometrium samples (six patients with two UL) replicated on two separate tissue microarrays. Average number of positively-stained nuclei was significantly higher in UL than myometrial tissue (t = 2.60, degrees of freedom (df) = 39, P = 0.01; paired t-test). **b)** Nuclear expression of FOXO1 is 2.32-fold greater in all UL compared to myometrial samples. Percent of positive nuclei were quantified in 335 UL and 35 myometrium samples (one myometrium sample failed) replicated on two separate tissue microarrays. Average number of positively-stained nuclei was significantly higher in UL than in myometrial tissue (t = 7.22, df = 56, $P = 1.52 \times 10^{-9}$; Welch's t-test). Error bars represent standard error (SE).

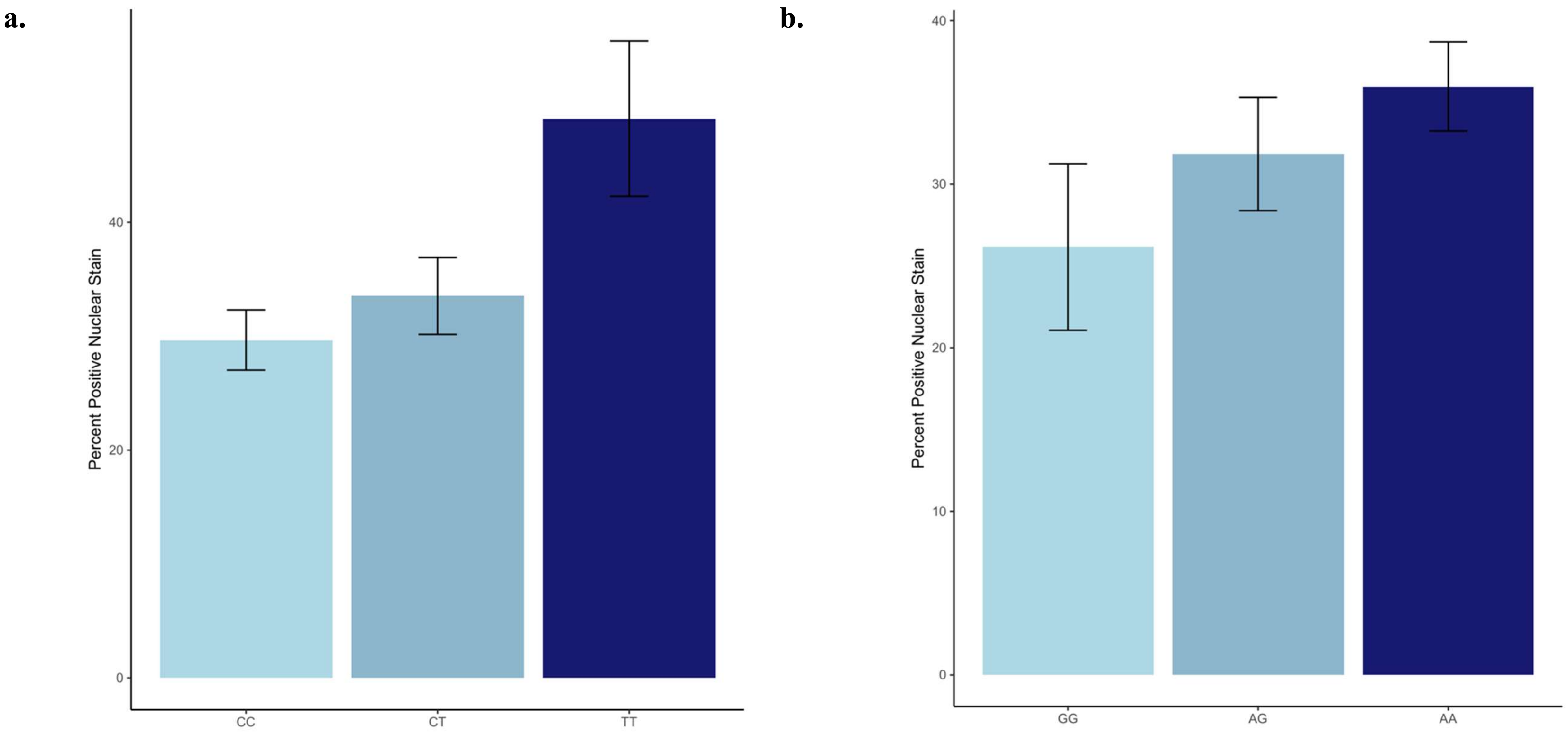

Supplementary Figure 13. Stratification of nuclear FOXO1 expression by genotype. FOXO1 expression and genotypes were quantified and determined in a total of 109 UL. **a)** One-way analysis of variance showed a significant relationship between allelic dosage of the rs6563799 risk variant [T] and FOXO1 expression (F = 3.2, df = 2, P = 0.047; one-way analysis of variance test). To compare mean expression of UL homozygous for the risk variant [T] against those with C/C or C/T genotypes, we performed an unpaired t-test (t = 2.51, df = 8, P = 0.035; Welch's t-test). **b)** One-way analysis of variance showed no significant relationship between allelic dosage of the rs7986407 risk variant [A] and FOXO1 expression (F = 1.5, df = 2, P = 0.22; one-way analysis of variance test). To compare mean expression of UL homozygous for the risk variant against those with G/G or G/A genotypes, we performed an unpaired t-test (t = 1.49, df = 105, P = 0.14; Welch's t-test). Error bars represent standard error (SE).

Supplementary Table 1. GWAS Cohorts. Four conventional population-based cohorts and one direct-to-consumer cohort from the FibroGENE consortium were included in the genome-wide association analyses.

Cohorts	Cases[a]	Controls[a]	Total
**Population-Based Cohorts**	20,406	223,918	244,324
Women's Genome Health Study (WGHS)	3,375	9,465	12,840
Northern Finland Birth Cohort (NFBC)	363	5,000	5,363
Queensland Institute of Medical Research (QIMR)	1,484	3,701	5,185
UK Biobank (UKBB)	15,184	205,752	220,936
**Direct-to-Consumer Cohort**			
23andMe	15,068	43,587	58,655
**Total**	35,474	267,505	302,979

[a] Cases and controls defined solely based on self-report or clinically documented history of uterine leiomyomata

Supplementary Table 2. Statistics of GWAS Cohorts. Overview of genomic inflation factor (λ_{GC}) used for adjustments and total number of SNPs analyzed in the GWA analyses.

**Cohort**	λ_{GC}	N_{SNP}
**WGHS**	1.008	8,767,907
**NFBC**	1.006	8,424,735
**QIMR**	0.998	8,279,309
**UKBB**	1.061	10,308,721
**23andMe**	1.051	9,164,495
**Meta-Analysis**	1.042	8,662,096

Supplementary Table 3. Imputation quality for lead SNPs with significant associations ($P < 5 \times 10^{-8}$) at 29 independent loci in UL GWAS meta-analysis. INFO scores describe the measure of imputation quality. 'GENOTYPED' indicates that the variant was directly genotyped in the corresponding cohort; 'n.i.' indicates that not data -- genotyped or imputed -- was available for that variant in the corresponding cohort.

		WGHS		NFBC		QIMR		UKBB		23andMe	
**Locus**	**rsID**	**INFO**	**MAF**	**INFO**	**MAF**	**INFO**	**MAF**	**INFO**	**MAF**	**INFO**	**MAF**
**1p36.12**	rs7412010	0.94434	0.160938	0.98788	0.17468	0.79977	0.164708	0.997947	0.162479	0.97199	0.14932
**2p23.2**	rs55819434	0.93637	0.097619	0.988051	0.102679	0.92683	0.0928901	0.988265	0.087571	0.97894	0.0998
**2p25.1**	rs35417544	0.85176	0.29865	0.947951	0.215019	0.88924	0.298684	0.995137	0.295729	0.96491	0.2958
**3q26.2**	rs35446936	0.80532	0.24715	0.995309	0.280215	0.9635	0.246264	0.99879	0.244179	0.97439	0.24074
**4q12**	rs62323682	0.87481	0.070863	0.872446	0.049243	0.80556	0.0595418	0.969738	0.067232	0.95829	0.07282
**4q13.3**	rs12640488	0.86236	0.461705	0.977749	0.431138	0.94592	0.491291	0.999451	0.497847	0.9572	0.47385
**4q22.3**	rs4699299	0.95468	0.301443	0.973472	0.25659	0.99289	0.315536	GENOTYPED	0.318079	0.97339	0.30409
**5p15.33**	rs72709458	0.66874	0.205772	0.861356	0.239415	0.6743	0.215104	0.974364	0.206267	0.8571	0.21732
**5q35.2**	rs2456181	0.93236	0.494745	0.979771	0.499736	n.i.	n.i.	0.994062	0.483581	0.99184	0.4987
**6p21.31**	rs116251328	0.65861	0.024194	0.981006	0.024827	0.61456	0.0188929	0.921527	0.026267	0.92843	0.03885
**6q25.2**	rs58415480	0.94367	0.158498	0.973945	0.219092	0.86195	0.155414	0.985055	0.154909	0.94393	0.15039
**7q31.2**	rs2270206	0.96986	0.154708	0.990408	0.156407	0.8504	0.152095	0.974707	0.151558	0.98598	0.15327
**9p24.3**	rs10976689	0.93506	0.385296	0.993636	0.47047	0.94897	0.401725	0.998079	0.401425	0.97902	0.3961
**10q24.3**	rs9419958	0.99979	0.142927	GENOTYPED	0.113649	0.99201	0.127586	0.998212	0.130176	GENOTYPED	0.14322
**10p11.22**	rs10508765	0.99994	0.208171	GENOTYPED	0.282118	0.99993	0.205402	0.994618	0.2007	GENOTYPED	0.20425
**11p15.5**	rs547025	0.96586	0.077661	0.996517	0.080207	0.63909	0.0782108	0.997458	0.074465	GENOTYPED	0.07628
**11p14.1**	rs11031006	0.94098	0.144334	0.996416	0.16973	0.84332	0.130768	0.992725	0.143276	GENOTYPED	0.14073
**11p13**	rs61889186	0.94592	0.15258	0.971734	0.079373	0.92577	0.161386	0.995959	0.15554	0.99681	0.15667
**11p13**	rs2785202	0.98905	0.443214	0.998134	0.467706	0.97537	0.446355	0.991613	0.450396	0.99727	0.44232
**11q22.3**	rs149934734	0.84897	0.020111	0.814485	0.01301	0.69313	0.0223896	0.988061	0.026191	0.95792	0.0257
**12q13.11**	rs2131371	0.99082	0.312061	0.999626	0.327552	0.97487	0.296947	0.997775	0.298335	0.9988	0.31133
**12q15**	rs11178393	0.91032	0.105642	0.974709	0.09203	0.79745	0.103151	0.982759	0.111854	0.98872	0.10904
**12q24.31**	rs28583837	0.56423	0.191796	0.997118	0.223943	0.67964	0.1892	0.996967	0.204049	0.96518	0.20816
**13q14.11**	rs117245733	0.41626	0.013512	0.904431	0.027631	0.53862	0.0155142	0.907964	0.01636	0.76043	0.01667
**17p13.1**	rs78378222	0.81404	0.011791	0.942854	0.019343	0.78132	0.0107054	0.954826	0.012367	0.95646	0.01119
**20p12.3**	rs16991615	0.99513	0.061616	0.867384	0.021527	0.70412	0.0635283	GENOTYPED	0.065107	GENOTYPED	0.06597
**22q13.1**	rs4821939	0.99163	0.206322	0.999306	0.239176	0.85259	0.22319	0.999092	0.212961	0.99985	0.20758
**Xp26.2**	rs12392108	0.98714	0.308941	n.i.	n.i.	0.96749	0.305922	0.994879	0.310589	0.99155	0.30733
**Xq13.1**	rs4360450	0.96241	0.352114	n.i.	n.i.	0.79191	0.332862	0.958791	0.354039	0.9627	0.34817

Supplementary Table 4. Secondary association signals from GCTA conditional analysis based on summary statistics of UL meta-analysis including all cases and an independent UKBB-based independent sample (N = 5,000) reference to calculate LD.

SNP	Chr	Position (bp)	RA	RAF	BETA	SE	*P*	Freq_ref	BETA_cond	SE_cond	*P* _cond
rs3951242	2	11306978	A	0.4697	-0.0461	0.0083	3.04E-08	0.462754	-0.0412056	0.00833378	7.64E-07
rs35417544	2	11680403	T	0.7058	0.0812	0.009	2.32E-19	0.71206	0.0765001	0.0090374	2.57E-17
rs62115045	2	12102123	A	0.1836	0.056	0.0104	7.17e-08	0.189487	0.0555765	0.0104065	9.2672e-08
rs17021300	4	94959212	A	0.0431	-0.0803	0.0202	7.06E-05	0.0440352	-0.0912414	0.020287	6.87E-06
rs4699299	4	95501166	T	0.6903	-0.0477	0.0087	4.72e-08	0.6882	-0.0513095	0.00873791	4.30E-09
rs11735529	4	53858948	C	0.5938	-0.0517	0.0109	2.20E-06	0.591331	-0.0514661	0.0109016	2.35E-06
rs62323682	4	54550174	T	0.9305	-0.1411	0.0163	4.92E-18	0.936551	-0.140909	0.016305	5.52E-18
rs72709458	5	1283755	T	0.2114	0.0983	0.0104	4.66E-21	0.202669	0.0812037	0.0110663	2.1693e-13
rs2853676	5	1288547	T	0.2705	0.0681	0.0091	8.16e-14	0.2743	0.0438914	0.00968141	5.80E-06
rs58415480	6	152562271	C	0.8439	-0.1748	0.0112	1.86E-54	0.847214	-0.166473	0.0112933	3.52E-49
rs6901631	6	152567047	T	0.8634	0.0946	0.0119	2.26E-15	0.869201	0.0732736	0.0119906	9.90E-10
rs10815466	9	680714	A	0.1509	0.0798	0.0114	2.17E-12	0.139617	0.0785269	0.0114035	5.73E-12
rs10976689	9	804886	A	0.598	-0.0608	0.0083	2.37e-13	0.602605	-0.0599155	0.00830273	5.34E-13
rs11031006	11	30226528	A	0.1432	-0.0925	0.0118	5.65E-15	0.145498	-0.0906666	0.0118053	1.59E-14
rs12224688	11	32327017	T	0.2405	0.0242	0.0096	0.01144	0.246392	0.0528699	0.00990759	9.49E-08
rs61889186	11	32367570	C	0.8457	-0.1167	0.0112	1.39E-25	0.843306	-0.130454	0.0115638	1.63E-29
rs2732552	11	35084592	T	0.447	-0.0608	0.0081	7.40E-14	0.448328	-0.0603466	0.00810189	9.44E-14
rs149934734	11	108315606	T	0.0255	0.2824	0.0259	1.10E-27	0.0242339	0.269216	0.0259789	3.66E-25
rs72993806	11	108359689	C	0.847	0.0895	0.0114	4.84E-15	0.845445	0.0810161	0.0114321	1.37E-12
rs11246003	11	213723	T	0.9583	0.1283	0.0203	2.74E-10	0.960892	0.13601	0.0203261	2.21E-11
rs547025	11	232855	T	0.9241	0.1189	0.0155	1.45E-14	0.923824	0.123838	0.0155212	1.48E-15
rs9548898	13	40300545	A	0.5109	0.0478	0.0081	3.22E-09	0.514798	0.0390599	0.00816714	1.73E-06
rs9548980	13	40495461	T	0.413	-0.0422	0.0083	4.17E-07	0.415591	-0.0378535	0.00837627	6.21E-06
rs117245733	13	40723944	A	0.0171	0.2614	0.0348	5.69E-14	0.0141443	0.253935	0.0348884	3.38E-13
rs6563812	13	40843805	C	0.4791	-0.0478	0.0083	7.88E-09	0.480718	-0.0437075	0.00831509	1.47E-07
rs7986407	13	41179798	A	0.6908	-0.064	0.0087	2.02E-13	0.683847	-0.0598016	0.00871211	6.69E-12

Footnote: Chr, Chromosome; Genomic position is shown related to GRCh37 (hg19); RA, risk allele; RAF, risk allele frequency; BETA, SE, *P*, effect size, standard error and *P*-value from UL final GWAS meta-analysis; Freq_ref, frequency of the risk allele in the reference sample; BETA_cond, SE_cond, *P*_cond, effect size, standard error and *P*-value from conditional analyses.

Supplementary Table 5. Fine-mapping results. A summary of the 99% credible sets for each index SNP identified as an independent signal originating from the 29 genomic loci associated with UL.

Index SNP	Number of SNPs	Interval (bp)	Start (bp)	End (bp)
rs10508765	35	56,512	31,913,890	31,970,401
rs10815466	4	3,447	680,714	684,160
rs10976689	13	7,388	804,231	811,618
rs11031006	22	156,355	30,215,261	30,371,615
rs11178393	17	27,874	71,145,532	71,173,405
rs11246003	18	39,108	202,017	241,124
rs116251328	67	286,218	34,094,919	34,381,136
rs117245733	1	1	40,723,944	40,723,944
rs11735529	2499	1,998,223	53,551,427	55,549,649
rs12224688	6044	6,854,352	29,228,649	36,083,000
rs12640488	22	123,121	70,524,658	70,647,778
rs149934734	4	213,682	108,143,456	108,357,137
rs16991615	1	1	5,948,227	5,948,227
rs17021300	3756	1,999,894	94,501,093	96,500,986
rs2131371	70	73,721	46,783,653	46,857,373
rs2270206	35	30,794	116,882,774	116,913,567
rs2456181	51	142,714	176,323,298	176,466,011
rs2732552	29	26,149	35,072,118	35,098,266
rs2853676	6301	1,999,736	284,117	2,283,852
rs35417544	17	40,484	11,662,178	11,702,661
rs35446936	50	114,361	169,477,506	169,591,866
rs3951242	543	1,957,414	10,682,491	12,639,904
rs4699299	40	148,050	95,447,259	95,595,308
rs4821939	25	191,290	40,529,415	40,720,704
rs547025	24	67,925	197,557	265,481
rs55819434	53	832,695	27,598,097	28,430,791
rs58415480	2	8,258	152,554,014	152,562,271
rs61889186	9	23,547	32,346,834	32,370,380
rs62115045	35	464,033	11,660,615	12,124,647
rs62323682	14	49,637	54,500,538	54,550,174
rs6563812	1139	1,996,133	39,725,173	41,721,305
rs6901631	11	53,131	152,538,985	152,592,115
rs72709458	3	3,966	1,279,790	1,283,755
rs72993806	30	382,360	107,983,096	108,365,455
rs7412010	8	47,731	22,422,721	22,470,451
rs78378222	1	1	7,571,752	7,571,752
rs7986407	106	291,089	41,143,720	41,434,808
rs9419958	8	12,779	105,674,854	105,687,632
rs9548898	1507	1,996,501	39,724,805	41,721,305
rs9548980	2373	1,997,175	39,724,131	41,721,305
rs28583837	199	462,669	123,450,765	123,913,433
rs12392108	31	164,705	69,976,939	70,141,643
rs4360450	19	80,267	131,251,326	131,331,592

Supplementary Table 6. Lead SNPs of UL GWAS meta-analysis compared to UKBB based UL, UL-limited-by-HMB and HMB GWAS results. Three out of 29 UL GWAS lead SNPs show genome-wide significance in UL-limited-by-HMB GWAS and are colored in red. Also, five UL GWAS lead SNPs show increased ORs in the UL-limited-by-HMB GWAS and are colored in green.

SNP	CHR	POS	A1	A1F	UL GWAS meta-analysis		UL GWAS in UKBB		UL-limited-to-HMB GWAS in UKBB		HMB GWAS in UKBB	
					P-UL	OR-UL (95% CI)	*P*-UL	OR-UL (95% CI)	*P*-UL/HMB	OR-UL/HMB (95% CI)	*P*-HMB	OR-HMB (95% CI)
rs7412010	1	22436446	C	0.16	2.43x10^{-29}	1.13 (1.11-1.16)	1.00x10^{-15}	1.14 (1.10-1.17)	3.70x10^{-2}	1.06 (1.00-1.13)	2.30x10^{-4}	1.09 (1.04-1.14)
**rs35417544**	**2**	**11680403**	**T**	**0.70**	**2.32x10^{-19}**	**1.08 (1.07-1.10)**	**4.80x10^{-9}**	**1.08 (1.05-1.11)**	**4.60x10^{-5}**	**1.10 (1.05-1.16)**	**3.60x10^{-3}**	**1.06 (1.02-1.09)**
**rs55819434**	**2**	**28333109**	**A**	**0.91**	**5.59x10^{-9}**	**0.92 (0.90-0.95)**	**4.80x10^{-7}**	**0.90 (0.86-0.94)**	**8.30x10^{-4}**	**0.88 (0.81-0.95)**	**1.10x10^{-2}**	**0.93 (0.88-0.98)**
rs35446936	3	169486508	A	0.24	1.03x10^{-8}	0.95 (0.93-0.97)	9.30x10^{-11}	0.91 (0.89-0.94)	4.10x10^{-4}	0.91 (0.87-0.96)	2.80x10^{-3}	0.94 (0.91-0.98)
rs62323682	4	54550174	T	0.93	4.92x10^{-18}	0.87 (0.84-0.90)	4.90x10^{-12}	0.85 (0.81-0.89)	7.80x10^{-4}	0.86 (0.79-0.94)	0.19	0.96 (0.90-1.02)
rs12640488	4	70600738	A	0.50	4.00x10^{-14}	0.94 (0.92-0.96)	2.30x10^{-9}	0.93 (0.91-0.95)	6.70x10^{-4}	0.93 (0.89-0.97)	0.68	0.99 (0.96-1.03)
rs4699299	4	95501166	T	0.68	4.72x10^{-8}	0.95 (0.94-0.97)	2.40x10^{-4}	0.95 (0.93-0.98)	0.51	0.98 (0.94-1.03)	0.63	0.99 (0.96-1.03)
**rs72709458**	**5**	**1283755**	**T**	**0.21**	**4.66x10^{-21}**	**1.10 (1.08-1.13)**	**5.20x10^{-15}**	**1.12 (1.09-1.16)**	**3.50x10^{-8}**	**1.16 (1.11-1.23)**	**1.80x10^{-8}**	**1.13 (1.08-1.17)**
**rs2456181**	**5**	**176450837**	**C**	**0.52**	**1.14x10^{-11}**	**0.94 (0.93-0.96)**	**3.70x10^{-9}**	**0.93 (0.91-0.95)**	**4.20x10^{-10}**	**0.87 (0.83-0.91)**	**6.40x10^{-5}**	**0.94 (0.91-0.97)**
rs116251328	6	34177510	A	0.03	2.95x10^{-8}	1.15 (1.09-1.21)	0.01	1.10 (1.02-1.19)	0.39	1.06 (0.92-1.23)	3.70x10^{-3}	1.17 (1.05-1.30)
rs58415480	6	152562271	C	0.85	1.86x10^{-54}	0.84 (0.82-0.86)	2.40x10^{-28}	0.83 (0.81-0.86)	1.20x10^{-3}	0.91 (0.85-0.96)	0.21	0.97 (0.93-1.02)
rs2270206	7	116913567	A	0.15	4.64x10^{-8}	1.06 (1.04-1.09)	0.01	1.04 (1.01-1.08)	0.86	1.00 (0.94-1.06)	0.78	1.01 (0.96-1.05)
rs10976689	9	804886	A	0.60	2.37x10^{-13}	0.94 (0.93-0.96)	1.40x10^{-19}	0.90 (0.88-0.92)	4.40x10^{-6}	0.90 (0.86-0.94)	0.05	0.97 (0.94-1.00)
rs10508765	10	31968783	A	0.80	1.51x10^{-10}	1.07 (1.05-1.09)	3.70x10^{-6}	1.07 (1.04-1.10)	1.10x10^{-2}	1.07 (1.02-1.13)	1.50x10^{-3}	1.07 (1.03-1.11)
rs9419958	10	105675946	T	0.13	1.05x10^{-16}	1.10 (1.08-1.13)	6.50x10^{-9}	1.11 (1.07-1.15)	1.70x10^{-3}	1.11 (1.04-1.19)	0.51	1.02 (0.97-1.07)
**rs547025**	**11**	**232855**	**T**	**0.93**	**1.45x10^{-14}**	**1.13 (1.09-1.16)**	**4.20x10^{-12}**	**1.17 (1.12-1.22)**	**1.70x10^{-6}**	**1.22 (1.13-1.34)**	**9.70x10^{-3}**	**1.09 (1.02-1.16)**
**rs11031006**	**11**	**30226528**	**A**	**0.14**	**5.65x10^{-15}**	**0.91 (0.89-0.93)**	**1.10x10^{-4}**	**0.93 (0.91-0.97)**	**9.80x10^{-5}**	**0.89 (0.83-0.94)**	**1.50x10^{-13}**	**0.84 (0.80-0.88)**
rs61889186	11	32367570	C	0.84	1.39x10^{-25}	0.89 (0.87-0.91)	5.10x10^{-18}	0.87 (0.84-0.90)	4.10x10^{-4}	0.90 (0.84-0.95)	0.04	0.95 (0.91-1.00)
rs2785202	11	35084835	C	0.55	6.94x10^{-14}	1.06 (1.04-1.08)	5.50x10^{-9}	1.07 (1.05-1.10)	9.40x10^{-4}	1.08 (1.03-1.13)	5.00x10^{-3}	1.05 (1.01-1.08)
**rs149934734**	**11**	**108315606**	**T**	**0.03**	**1.10x10^{-27}**	**1.33 (1.26-1.40)**	**9.50x10^{-20}**	**1.41 (1.31-1.51)**	**5.50x10^{-8}**	**1.45 (1.28-1.69)**	**0.09**	**1.09 (0.99-1.21)**
rs2131371	12	46796522	A	0.30	1.62x10^{-18}	0.93 (0.91-0.94)	1.20x10^{-10}	0.92 (0.90-0.94)	5.60x10^{-4}	0.92 (0.88-0.96)	0.64	0.99 (0.96-1.03)
rs11178393	12	71150658	T	0.89	3.34x10^{-8}	1.08 (1.05-1.10)	5.90x10^{-4}	1.07 (1.03-1.11)	7.10x10^{-2}	1.07 (0.99-1.14)	0.78	0.99 (0.94-1.05)
rs28583837	12	123863620	A	0.20	2.31x10^{-8}	0.94 (0.92-0.96)	3.60x10^{-7}	0.93 (0.90-0.96)	1.30x10^{-2}	0.93 (0.88-0.98)	0.02	0.95 (0.91-0.99)
rs117245733	13	40723944	A	0.02	5.69x10^{-14}	1.30 (1.21-1.39)	8.60x10^{-9}	1.33 (1.20-1.46)	2.00x10^{-3}	1.32 (1.11-1.59)	0.74	0.98 (0.86-1.12)
rs78378222	17	7571752	T	0.99	7.11x10^{-31}	0.65 (0.60-0.70)	1.00x10^{-27}	0.55 (0.49-0.61)	2.40x10^{-7}	0.59 (0.48-0.72)	4.80x10^{-4}	0.76 (0.66-0.89)
**rs16991615**	**20**	**5948227**	**A**	**0.07**	**8.82x10^{-10}**	**1.11 (1.07-1.14)**	**4.90x10^{-7}**	**1.13 (1.08-1.18)**	**7.00x10^{-5}**	**1.19 (1.10-1.31)**	**3.00x10^{-8}**	**1.21 (1.13-1.29)**
rs4821939	22	40659251	A	0.21	7.83x10^{-16}	1.08 (1.06-1.10)	3.50x10^{-10}	1.10 (1.07-1.13)	5.10x10^{-5}	1.10 (1.04-1.16)	0.25	1.02 (0.98-1.07)
rs12392108	X	131314262	A	0.31	5.87x10^{-46}	1.13 (1.11-1.15)	1.40x10^{-22}	1.13 (1.11-1.16)	4.90x10^{-7}	1.13 (1.08-1.19)	3.30x10^{-3}	1.05 (1.02-1.09)
rs4360450	X	70146398	A	0.35	2.06x10^{-18}	1.08 (1.06-1.10)	1.70x10^{-15}	1.11 (1.08-1.13)	1.20x10^{-4}	1.09 (1.05-1.15)	2.90x10^{-4}	1.07 (1.03-1.10)

Supplementary Table 7. Results of Mendelian randomization. The inverse-variance-weighted (IVW) model was used to test causality between exposure and outcome, IVW (Q) method for heterogeneity in estimates, and MR Egger for horizontal pleiotropy.

	**IVW**			**Heterogeneity (IVW)**			**Horizontal pleiotropy (MR Egger)**			**MR-PRESSO**		
	β	s.e.	*P*	*Q*	df	*P*	intercept	s.e.	*P*	β	s.d.	*P*
**UL (exposure) > HMB (outcome)**	0.26	0.04	$1.2e^{-12}$	17.54	12	0.13	0.01	0.01	0.36			
**Endometriosis (exposure) > UL (outcome)**	0.36	0.12	$3.7e^{-3}$	361.38	15	$9.5e^{-68}$	-0.09	0.05	0.10	0.29	0.07	0.002
**UL (exposure) > endometriosis (outcome)**	0.13	0.14	0.37	19.47	21	0.55	-0.01	0.03	0.68			

Supplementary Table 8. Linkage disequilibrium between the UL and previously reported endometriosis lead SNPs at 1p36.12, 2p25.1, 6q25.2, and 11p14.1 in women of European ancestry.

Chr location	UL lead SNP	RA	RAF_{EUR}	Endometriosis lead SNP[a]	RA	RAF_{EUR}	r^2
1p36.12	rs7412010	C	0.15	rs12037376	A	0.17	0.90
2p25.1	rs35417544	T	0.69	rs11674184	T	0.61	0.21
6q25.2	rs58415480	C	0.84	rs71575922	G	0.16	0.91
11p14.1	rs11031006	A	0.14	rs74485684	T	0.84	0.81

RA, risk allele; RAF_{EUR}, average risk allele frequency in European samples
[a] Sapkota *et al.* Nat Commun 2016

Supplementary Table 9. Demographic overview of NHSII, WHS, and UKBB cohorts at baseline.

	NHSII	WHS	UKBB
**Age (Mean [Standard deviation])**	36.1 (4.6)	53.6 (6.2)	56.3 (8.0)
**Ancestry (%)**			
White	91.4	94.8	94.2
Black	1.5	1.8	1.7
Asian	2.0	1.3	2.0
Hispanic	1.5	1.0	Not available
Other	3.5	0.2	1.6
**BMI (%)**			
<20	16.0	5.1	3.4
20-21.9	27.2	16.1	9.7
22-23.9	20.2	20.7	16.8
24-24.9	7.6	10.4	9.5
25-26.9	9.9	15.5	17.6
27-29.9	8.3	15.2	19.0
≥30	10.8	17.1	23.5
**Smoking History (%)**			
Never	66.0	53.1	44.6
Past	21.1	36.5	20.2
Current	12.9	10.3	6.8
**Age at Menarche in Years (%)**			
<11	7.4	8.3	4.5
11	16.4	16.3	14.9
12	30.2	28.5	18.3
13	27.6	28.9	23.6
14-15	15.0	14.7	29.7
>15	3.4	3.2	5.7
**Oral Contraceptive Use (%)**			
Never	17.4	27.5	18.8
Ever	82.6	72.5	80.6
**Parity (%)**			
Nulliparous	31.3	12.7	18.7
1	19.2	9.0	13.3
2	31.9	30.8	43.6
3	13.6	24.3	17.7
≥4	4.0	22.9	6.4
**Age at First Birth (%)**			
<25	38.6	59.6	30.5
25-29	45.7	28.7	21.1
>29	15.7	11.6	11.8

Values of polytomous variables may not sum to 100% due to rounding.

Supplementary References

1. Eggert SL, Huyck KL, Somasundaram P, et al. Genome-wide linkage and association analyses implicate FASN in predisposition to uterine leiomyomata. Am J Hum Genet 91, 621-628 (2012).

2. Treloar SA, Wicks J, Nyholt DR, et al. Genomewide linkage study in 1,176 affected sister pair families identifies a significant susceptibility locus for endometriosis on chromosome 10q26. Am J Hum Genet 77, 365-76 (2005).

3. Treloar SA, Do KA, O'Connor VM, O'Connor DT, Yeo MA, Martin NG. Predictors of hysterectomy: an Australian study. Am J Obstet Gynecol 180, 945-54 (1999).

4. Sudlow C, Gallacher J, Allen N, et al. UK biobank: an open access resource for identifying the causes of a wide range of complex diseases of middle and old age. PLoS Med 12, e1001779 (2015).

5. Marshall, L.M. et al. Variation in the incidence of uterine leiomyoma among premenopausal women by age and race. Obstet Gynecol 90, 967-73 (1997).

6. Missmer SA, Hankinson SE, Spiegelman D, et al. Incidence of laparoscopically confirmed endometriosis by demographic, anthropometric, and lifestyle factors. Am J Epidemiol 160, 784-9 (2004).
