# Supplementary material for: Genome-wide association and epidemiological analyses reveal common genetic origins between uterine leiomyomata and endometriosis
Source: Nat Commun. 2019 Oct 24;10:4857. doi: 10.1038/s41467-019-12536-4 (PMC6813337; doi:10.1038/s41467-019-12536-4)
Supplement: Supplementary file 1 — Supplementary Information [file 41467_2019_12536_MOESM1_ESM.pdf]

Reviewers' comments:

Reviewer #1 (Remarks to the Author):

Uterine leiomyomata (UL) (or uterine fibroids) is a common neoplasm that is associated with several conditions including infertility and endometriosis and often results in hysterectomy. Clues to the biology of this condition are further revealed in the submitted manuscript focused on results of a GWAS meta-analysis. Over 35,000 cases and over 260,000 controls of white European ancestry were included from four large cohort studies and other available data. Strengths of this report include the use of additional phenotypic data available in some datasets. The analytic approaches that were employed provide a thorough evaluation of the genetic relationships for UL, HMB and endometriosis. The main findings of this report are eight novel genome-wide significant loci associated with UL and upon further stratification, three loci were found to be associated with heavy menstrual bleeding (HMB). The analysis of HMB is a particular strength for insights into treatment targets. The epidemiologic studies provided evidence for genetic basis for the association between endometriosis and UL.

The significance of these findings are that they may lead to pharmacologic intervention as well a better understanding of the relationship between UL and endometriosis. Although this is a very strong paper with important biologic relevance, there is some disappointment that the authors were unable to evaluate associations among women of African ancestry who have the highest prevalence of uterine fibroids. Therefore, we cannot evaluate the generalizability of the findings to this population.

Reviewer #3 (Remarks to the Author):

Gallagher and colleagues report the largest GWAS meta-analysis for susceptibility to uterine leiomyomata (UL), a common condition associated with extensive morbidity. They use state-of-the-art genetic and epidemiological methods to characterise the relationship between UL and endometriosis. They also explore associations with heavy menstrual bleeding with a view to identifying potential therapeutic targets specific to this symptom. There are, however, some concerns in their analysis that I would prefer that the authors address clearly.

1. How many of the lead SNPs in Table 1 were genotyped and how many were imputed? What was the imputation quality for the imputed lead SNPs? If quality was < 0.8 for some of these SNPs, did they have genotyped or better imputed and genome-wide significant LD-proxies available? I could not find this information in the manuscript.

2. Related to #1, what is the imputation quality for the lead SNPs marked in Figure 2, particularly in panels a and c? If these are not genotyped SNPs, and considering the recombination peaks and SNP LD architecture shown, have the authors ruled out the possibility that these genome-wide significant singleton driver SNPs are not merely artifacts due to poor imputation quality? I am concerned: > 0.4 may be an adequate cut-off genome-wide but it is certainly safer to impose a threshold of > 0.8 for the lead SNPs, particularly singletons such as those shown.

3. To what extent are the Mendelian Randomisation analyses reported in the manuscript affected by bias due to control sample overlap? (for a discussion on this please see: Burgess et al., Bias due to participant overlap in two-sample Mendelian randomization, Genetic Epidemiology 2016). For example, it appears that the UK BioBank was used for both the UL and HMB GWAS. Alternative approaches may involve performing single-sample MR in UKBB or adjusting summary statistic standard errors that are

entered into the inverse-variance weighted model using the decoupling approach to account for correlation due to sample overlap (see Han et al., A general framework for meta-analyzing dependent studies with overlapping subjects in association mapping, HMG 2016).

4. Page 11, line 200: “MR suggests that genetic predisposition to endometriosis (exposure) is causally linked to an increased risk of UL (outcome); the b of 0.36 is significant (P = 3.7 x 10-3) in the IVW model (heterogeneity P = 9.5 x 10-68)”. The strong heterogeneity observed necessitates far greater caution in interpreting this result as evidence for causality and calls for substantial additional analyses. There are some approaches to identifying the minimum subset of SNPs (of the 21 endometriosis lead SNPs) that when used as a genetic instrument may eliminate heterogeneity (these methods are applied in Corbin et al., Diabetes. 2016;65:3002–7). It may also be possible to use the HEIDI outlier test in SMR for the same purpose – the authors use this for eQTL analyses but not in MR. And finally, it may be possible to apply www.biorxiv.org/content/10.1101/566851v1 to dissect whether particular loci, such as those harbouring hormone signalling genes, drive the underlying association. A leave-one-out plot from MR-Base (the R package used) would also be helpful to identify heterogeneous lead SNPs and inform on potentially homogeneous SNP subsets and related molecular mechanisms.

5. At the four genome-wide significant loci that overlap between endometriosis and UL, the authors discuss major candidate genes based on biology. However, there is no discussion of the fact mentioned in the previous paragraph that the direction of allelic effect at only one of the four loci appears to be consistent between the two diseases. Does this not complicate the direction and interpretation of the causal MR result and the potential importance of these genes?

Siddhartha Kar

Reviewer #1 (Remarks to the Author):

Uterine leiomyomata (UL) (or uterine fibroids) is a common neoplasm that is associated with several conditions including infertility and endometriosis and often results in hysterectomy. Clues to the biology of this condition are further revealed in the submitted manuscript focused on results of a GWAS meta-analysis. Over 35,000 cases and over 260,000 controls of white European ancestry were included from four large cohort studies and other available data. Strengths of this report include the use of additional phenotypic data available in some datasets. The analytic approaches that were employed provide a thorough evaluation of the genetic relationships for UL, HMB and endometriosis. The main findings of this report are eight novel genome-wide significant loci associated with UL and upon further stratification, three loci were found to be associated with heavy menstrual bleeding (HMB). The analysis of HMB is a particular strength for insights into treatment targets. The epidemiologic studies provided evidence for genetic basis for the association between endometriosis and UL.

The significance of these findings are that they may lead to pharmacologic intervention as well a better understanding of the relationship between UL and endometriosis. Although this is a very strong paper with important biologic relevance, there is some disappointment that the authors were unable to evaluate associations among women of African ancestry who have the highest prevalence of uterine fibroids. Therefore, we cannot evaluate the generalizability of the findings to this population.

The reviewer raises an important point. Women with African ancestry have higher prevalence, earlier onset, and larger and more numerous uterine fibroids than women with European ancestry. To date, we have co-authored one GWAS in African American women, revealing a genome-wide significant locus near *CYTH4* (cytohesin 4) on chromosome 22q13.1 (Hellwege et al. A multi-stage genome-wide association study of uterine fibroids in African Americans. Hum Genet. 2017). We did not observe a similar association in our GWAS meta-analysis, supporting that ancestral differences in predisposition to uterine fibroids exist. We wholeheartedly agree with the reviewer that it is of great importance in the future to perform GWAS on a larger set of women with African ancestry to understand better genomic predisposition to uterine fibroids in this population.

Reviewer #3 (Remarks to the Author):

Gallagher and colleagues report the largest GWAS meta-analysis for susceptibility to uterine leiomyomata (UL), a common condition associated with extensive morbidity. They use state-of-the-art genetic and epidemiological methods to characterise the relationship between UL and endometriosis. They also explore associations with heavy menstrual bleeding with a view to identifying potential therapeutic targets specific to this symptom. There are, however, some concerns in their analysis that I would prefer that the authors address clearly.

1. How many of the lead SNPs in Table 1 were genotyped and how many were imputed? What was the imputation quality for the imputed lead SNPs? If quality was < 0.8 for some of these SNPs, did they have genotyped or better imputed and genome-wide significant LD-proxies available? I could not find this information in the manuscript.

We thank the reviewer for this insightful comment. We have now added a new Supplementary Table (Supplementary Table 3) in the manuscript, which includes this previously missing information. All lead SNPs that we report were either directly genotyped or had remarkably high imputation quality (> 0.9) in at least two cohorts, with one exception: rs72709458 on chromosome 5. This lead SNP showed strong imputation quality in the UKBB (0.97) cohort, but reduced quality in the other four cohorts: WGHS (0.67), NFBC (0.86), QIMR (0.67), and 23andMe (0.86). Nonetheless, more than one variant in linkage with rs72709458 met our high-quality imputation criteria (genotyped or imputation quality > 0.9 in at least two cohorts).

2. Related to #1, what is the imputation quality for the lead SNPs marked in Figure 2, particularly in panels a and c? If these are not genotyped SNPs, and considering the recombination peaks and SNP LD architecture shown, have the authors ruled out the possibility that these genome-wide significant singleton driver SNPs are not merely artifacts due to poor imputation quality? I am concerned: > 0.4 may be an adequate cut-off genome-wide but it is certainly safer to impose a threshold of > 0.8 for the lead SNPs, particularly singletons such as those shown.

As mentioned above, we have added a new Supplementary Table (Supplementary Table 3) in the manuscript, including this previously missing information. In Figure 2 panel a, the lead SNP rs117245733 shows strong imputation quality in the NFBC (0.90) and UKBB (0.91) cohorts, while reduced quality in the other three cohorts: WGHS (0.42), QIMR (0.54) and 23andMe (0.76). In panel b, the lead SNP rs78378222 shows good imputation quality in WGHS (0.81), NFBC (0.94), UKBB (0.95) and 23andMe (0.96) cohorts, while slightly reduced quality in QIMR (0.78). In panel c, the lead SNP rs16991615 has been genotyped in two cohorts: UKBB and 23andMe. It also shows strong imputation quality in WGHS (1.00) and NFBC (0.87) cohorts, while reduced quality in QIMR (0.70). Thus, the lead SNPs in Figure 2 were either directly genotyped or had remarkably high imputation quality (> 0.9) in at least two cohorts. We have added a sentence to the Methods section describing the confirmation criteria for lead SNPs (page 19; line 412-414).

3. To what extent are the Mendelian Randomisation analyses reported in the manuscript affected by bias due to control sample overlap? (for a discussion on this please see: Burgess et al., Bias due to participant overlap in twosample Mendelian randomization, Genetic

Epidemiology 2016). For example, it appears that the UK BioBank was used for both the UL and HMB GWAS. Alternative approaches may involve performing single-sample MR in UKBB or adjusting summary statistic standard errors that are entered into the inverse-variance weighted model using the decoupling approach to account for correlation due to sample overlap (see Han et al., A general framework for meta-analyzing dependent studies with overlapping subjects in association mapping, HMG 2016).

The reviewer makes an important point. In our Mendelian Randomization analysis, where we assessed the causality of genetic association between UL (exposure) and endometriosis (outcome), WGHS was excluded from the UL GWAS to avoid any overlap between samples in the exposure and outcome cohorts. No sample overlap was present either in the reverse causation model.

For the Mendelian Randomization analysis on the causality of genetic association between UL (exposure) and HMB (outcome), we have now conducted a UL GWAS excluding all the HMB cases, which is consistent with the method described in Burgess et al. 2016, as suggested by the reviewer. Results of this experiment are described on page 8 (lines 181-184) in the revised manuscript and corresponding changes have been made to the Supplementary Table 7. Briefly, our analysis shows that genetic predisposition to UL is causally linked to an increased risk of HMB; the β estimate of 0.26 was significant in the inverse-variance-weighted (IVW) model (P = 1.2 x 10^{-12}) and showed no significant heterogeneity (P = 0.13). The MR Egger regression analyses showed no significant directional pleiotropy (intercept = 0.01, P = 0.36).

4. Page 11, line 200: "MR suggests that genetic predisposition to endometriosis (exposure) is causally linked to an increased risk of UL (outcome); the b of 0.36 is significant (P = 3.7 x 10-3) in the IVW model (heterogeneity P = 9.5 x 10-68)". The strong heterogeneity observed necessitates far greater caution in interpreting this result as evidence for causality and calls for substantial additional analyses. There are some approaches to identifying the minimum subset of SNPs (of the 21 endometriosis lead SNPs) that when used as a genetic instrument may eliminate heterogeneity (these methods are applied in Corbin et al., Diabetes. 2016;65:30027). It may also be possible to use the HEIDI outlier test in SMR for the same purpose the authors use this for eQTL analyses but not in MR. And finally, it may be possible to apply www.biorxiv.org/content/10.1101/566851v1 to dissect whether particular loci, such as those harbouring hormone signalling genes, drive the underlying association. A leave-one-out plot from MR-Base (the R package used) would also be helpful to identify heterogeneous lead SNPs and inform on potentially homogeneous SNP subsets and related molecular mechanisms.

As suggested by the reviewer, we have now added a leave-one out plot to the Supplementary Information (Supplementary Figure 8), as well as text of its results to the manuscript (page 9; lines 199-201). Leave-one-out sensitivity analysis shows that no single SNP alone drives the significant relationship between endometriosis and UL, but instead the relationship is accounted for by contributions from multiple variants across the genome. Nonetheless, due to the high degree of heterogeneity observed in our results, we have now leveraged a similar approach to the one published in Corbin et al. 2016, to identify the minimum set of variants that when used as a genetic instrument eliminate heterogeneity. As expected, the subset of variants was consistent with the results from our leave-one-out analysis. We have also added another leave-one out plot to the

Supplementary Information representing the minimum set of variants (Supplementary Figure 9). The results remain significant (P = 4.3 x 10^{-3}) in the absence of heterogeneity (P = 0.23), albeit with a reduced β estimate of 0.12 in the inverse-variance-weighted (IVW) model. Additionally, we applied the MR pleiotropy residual sum and outlier (MR-PRESSO; Verbanck et al. 2018) test to identify and adjust for variants causing significant bias through horizontal pleiotropy. Outlier-adjusted estimates still provide significant evidence for a causal estimate of endometriosis on UL (β = 0.281, P < 0.005). These results have now been added to the revised manuscript (page 9; lines 201-209).

We greatly appreciate the reviewer's attention to our Mendelian Randomization analyses as it has helped to refine the set of variants underlying the relationship between endometriosis and UL. Furthermore, we are keen to continue our work to characterize better the genetic instruments as more data and methods become available.

5. At the four genome-wide significant loci that overlap between endometriosis and UL, the authors discuss major candidate genes based on biology. However, there is no discussion of the fact mentioned in the previous paragraph that the direction of allelic effect at only one of the four loci appears to be consistent between the two diseases. Does this not complicate the direction and interpretation of the causal MR result and the potential importance of these genes?

Average age-of-onset for endometriosis is approximately 10 years prior to the average age-of-onset of UL (exact age of onset endo vs. exact age of onset UL). In the light of the temporal relationship between the two diseases, one interpretation of the results from our Mendelian Randomization analysis is that genetic predisposition to endometriosis is causally linked to an increased risk of UL. However, we also observe disagreement between the direction of allelic effects in UL and endometriosis in several instances. Therefore, an alternative, more cautious interpretation is that our Mendelian Randomization results indicate significant overlap in the underlying biology of the two diseases. Nonetheless, our epidemiologic follow-up work in WHS and UKBB cohorts indicates that there is a significant increase in the odds of co-reporting endometriosis and UL. Further, results from longitudinal analyses in the Nurses' Health Study II show women with a history of endometriosis are at significantly higher risk for UL when compared to unaffected women (hazard ratio of 1.56). Further work will be required to quantify the contribution of genetic effects to the directional relationship between endometriosis and UL and what portion of our Mendelian Randomization results reflect the fundamental pathobiological overlap in these two serious diseases of the uterus.

We have now added discussion related to this topic to the revised manuscript (pages 15-16, lines 346-352).

REVIEWERS' COMMENTS:

Reviewer #3 (Remarks to the Author):

The authors have adequately answered all my questions and I have no further concerns.

Siddhartha Kar
