# Supplementary material for: Genome-wide association and epidemiological analyses reveal common genetic origins between uterine leiomyomata and endometriosis
Source: Nat Commun. 2019 Oct 24;10:4857. doi: 10.1038/s41467-019-12536-4 (PMC6813337; doi:10.1038/s41467-019-12536-4)
Supplement: Supplementary file 2 — Peer Review File [file 41467_2019_12536_MOESM2_ESM.pdf]

nature research

Corresponding author(s): Cynthia C. Morton
Netta Mäkinen

Last updated by author(s): Aug 16, 2019

Reporting Summary

Nature Research wishes to improve the reproducibility of the work that we publish. This form provides structure for consistency and transparency in reporting. For further information on Nature Research policies, see Authors & Referees and the Editorial Policy Checklist.

Statistics

For all statistical analyses, confirm that the following items are present in the figure legend, table legend, main text, or Methods section.

n/a	Confirmed	
☐	☒	The exact sample size (*n*) for each experimental group/condition, given as a discrete number and unit of measurement
☐	☒	A statement on whether measurements were taken from distinct samples or whether the same sample was measured repeatedly
☐	☒	The statistical test(s) used AND whether they are one- or two-sided<br>*Only common tests should be described solely by name; describe more complex techniques in the Methods section.*
☐	☒	A description of all covariates tested
☐	☒	A description of any assumptions or corrections, such as tests of normality and adjustment for multiple comparisons
☐	☒	A full description of the statistical parameters including central tendency (e.g. means) or other basic estimates (e.g. regression coefficient) AND variation (e.g. standard deviation) or associated estimates of uncertainty (e.g. confidence intervals)
☐	☒	For null hypothesis testing, the test statistic (e.g. F, t, r) with confidence intervals, effect sizes, degrees of freedom and P value noted<br>*Give P values as exact values whenever suitable.*
☐	☒	For Bayesian analysis, information on the choice of priors and Markov chain Monte Carlo settings
☐	☒	For hierarchical and complex designs, identification of the appropriate level for tests and full reporting of outcomes
☐	☒	Estimates of effect sizes (e.g. Cohen's d, Pearson's r), indicating how they were calculated

Our web collection on statistics for biologists contains articles on many of the points above.

Software and code

Policy information about availability of computer code

Data collection	No software was used for data collection.
Data analysis	ShapeIt2, IMPUTE2, EIGENSTRAT, BOLT-LMM v.2.3.2, METAL, LDSC, GCTA, R Studio, DEPICT, SMR and Aperio ImageScope softwares were used to analyze data in this study.

For manuscripts utilizing custom algorithms or software that are central to the research but not yet described in published literature, software must be made available to editors/reviewers. We strongly encourage code deposition in a community repository (e.g. GitHub). See the Nature Research guidelines for submitting code & software for further information.

Data

Policy information about availability of data

All manuscripts must include a data availability statement. This statement should provide the following information, where applicable:

- Accession codes, unique identifiers, or web links for publicly available datasets
- A list of figures that have associated raw data
- A description of any restrictions on data availability

The authors declare that the data supporting the findings of this study are available within the article and its Supplementary Information files. UL GWAS meta-analysis summary statistics (without 23andMe), UL GWAS limited by HMB and HMB GWAS summary statistics will be made available through the NHGRI-EBI GWAS Catalog https://www.ebi.ac.uk/gwas/downloads/summary-statistics. To request access to 23andMe GWAS summary statistics, please visit https://research.23andme.com/dataset-access/.

Field-specific reporting

Please select the one below that is the best fit for your research. If you are not sure, read the appropriate sections before making your selection.

☒ Life sciences ☐ Behavioural & social sciences ☐ Ecological, evolutionary & environmental sciences

For a reference copy of the document with all sections, see nature.com/documents/nr-reporting-summary-flat.pdf

Life sciences study design

All studies must disclose on these points even when the disclosure is negative.

Sample size	No statistical methods were used to predetermine sample sizes. Sample sizes were chosen to be as large as possible. Adequate statistics and rigorous statistical thresholds (e.g. Bonferroni correction) have been applied throughout the manuscript in order to make sure that the observed effects are significant given the reported sample size.
Data exclusions	Genotyping data (quality control and imputation):<br>1. All cases and controls with a genotyping call rate < 0.98 were excluded from the study.<br>2. After imputation, SNPs with call rates of < 99% or with deviation from Hardy-Weinberg equilibrium were excluded from further analyses.<br>3. Also poorly imputed SNPs (r2 < 0.4) and SNPs with a MAF of < 1% were excluded.<br>4. All observed individual outliers were removed based on principal component analysis (PCA).<br><br>Meta-analysis:<br>1. SNPs present only in one GWAS study were excluded.<br>2. Heterogeneity in effect sizes across studies was tested using Cochran's Q-statistic and indicated when relevant.<br><br>FOXO1 immunohistochemistry:<br>Duplicates of tissue punches from the same samples were included on the tissue microarray (TMA). If either one of the two punches representing the same sample failed, we excluded the sample from further analyses.
Replication	We confirm 21 out of 26 genomic loci previously reported to be significantly associated with UL. We have not re-run the analyses with another independent set of participants; however, our analyses were performed in large sample sizes and assessment of effect size heterogeneity across cohorts was performed and indicated when relevant. For FOXO1 immunohistochemistry, biological replicates (i.e. individual punches) were combined into a single measurement by taking the mean.
Randomization	For UL GWA and epidemiological analyses, cases and controls were defined based on either self-reported or clinically documented UL or endometriosis history. For heavy menstrual bleeding GWAS, only cases with hospital-linked medical records documenting heavy menstrual bleeding were considered.
Blinding	No investigator blinding was applied or necessary during data acquisition or analyses.

Reporting for specific materials, systems and methods

We require information from authors about some types of materials, experimental systems and methods used in many studies. Here, indicate whether each material, system or method listed is relevant to your study. If you are not sure if a list item applies to your research, read the appropriate section before selecting a response.

Materials & experimental systems

n/a	Involved in the study	
☐	☒	Antibodies
☒	☐	Eukaryotic cell lines
☒	☐	Palaeontology
☒	☐	Animals and other organisms
☐	☒	Human research participants
☒	☐	Clinical data

Methods

n/a	Involved in the study	
☒	☐	ChIP-seq
☒	☐	Flow cytometry
☒	☐	MRI-based neuroimaging

Antibodies

Antibodies used	FoxO1 (C29H4) Rabbit mAb #2880, Cell Signaling Technology
Validation	According to the vendor, FoxO1 (C29H4) Rabbit mAb detects endogenous levels of total FoxO1 protein. The antibody does not detect exogenously expressed family members FoxO3a or FoxO4. For this study, FoxO1 antibody was first optimized at the Specialized Histopathology Core of the Dana-Farber/Harvard Cancer Center for immunohistochemistry. Immunohistochemistry was carried out using the BOND staining system (Leica Biosystems, Buffalo Grove, IL) resulting in a final antibody dilution 1:100.

FOXO1 immunostaining was performed on two replicate TMAs containing 335 UL and 36 myometrium tissue samples from 200 white women of European ancestry obtained from myomectomies and hysterectomies.

Human research participants

Policy information about studies involving human research participants

Population characteristics	The population characteristics of human research participants have been described in the original studies where human-associated samples were collected.
Recruitment	Recruitment of the human research participants has been described in the original studies where human-associated samples were collected.
Ethics oversight	Informed consent was obtained from all participants. Study protocols for each cohort have been approved by relevant ethical committees: the Partners HealthCare System Human Research Committee (WGH/WGHS), the Ethical Committee of the Northern Ostrobothnia Hospital District (NFBC), the Human Research Ethics Committee at the QIMR Berghofer Medical Research Institute and the Australian Twin Registry (QIMR), the North West Multi-centre Research Ethics Committee (UKBB), the Ethical and Independent Review Services (an external institutional review board; 23andMe), and the Institutional Review Boards at Harvard T.H. Chan School of Public Health and Brigham and Women's Hospital (Partners Human Research Committee) (NHSII).

Note that full information on the approval of the study protocol must also be provided in the manuscript.
